# Kinematics of strikes in venomous snakes

**DOI:** 10.1242/jeb.250347

**Published:** 2025-10-23

**Authors:** Silke G. C. Cleuren, James P. Rule, Remi Ksas, Anthony Herrel, David P. Hocking, Alistair R. Evans

**Affiliations:** ^1^ School of Biological Sciences, Monash University, Melbourne, VIC 3800, Australia; ^2^ Venomworld, 77400 Saint-Thibault-des-Vignes, France; ^3^ Mécanismes Adaptatifs et Evolution, UMR 7179, Muséum national d'Histoire naturelle CNRS, 75231 Paris, France; ^4^ Department of Biology, Evolutionary Morphology of Vertebrates, Ghent University, 9000 Ghent, Belgium; ^5^ Department of Biology, University of Antwerp, 2610 Wilrijk, Belgium; ^6^ Naturhistorisches Museum Bern, 3005 Bern, Switzerland; ^7^ Tasmanian Museum and Art Gallery, Hobart, TAS 7000, Australia; ^8^ Museums Victoria Research Institute, Melbourne, VIC 3001, Australia

**Keywords:** Viperidae, Elapidae, Colubridae, Kinematics, Biomechanics, High-speed video analysis

## Abstract

Venomous snakes owe their evolutionary success in part to the effectiveness of their strike. The success of a strike depends on reaching the prey quickly before it startles and has the chance to escape. Here, we present the first ever large-scale experiment comparing strike performance across 36 venomous snake species from three families (31 Viperidae, 4 Elapidae and 1 Colubridae). We used two high-speed video cameras (1000 frames s^−1^) to capture strikes at a ballistics gel prey and tracked the strike trajectory in three dimensions. The 3D coordinates were used to measure strike kinematics and performance. Kinematic performance was compared within Viperidae across predation style, diel activity pattern, diet, habitat type, temperature and first jaw contact with prey. Kinematic variables (peak velocity, peak acceleration, gape angle, start distance, contact angle, head size) varied by the part of the jaw that first contacts the prey. Start distance to prey also varied by peak acceleration, jaw gape angle and contact angle with prey. Vipers typically reached higher peak velocities than elapids; however, some elapids such as *Acanthophis rugosus* reached equally high velocities. Peak velocities were found to be higher in ambush predators and in snakes that prey on mammalian prey. Prey was often reached within 100 ms, which falls within the mammalian startle response. Behavioural differences across the three families were also observed: Viperidae performed a smooth strike that was often followed by fang repositioning; Elapidae reached their prey quickly, bit and repeatedly squeezed prey with their jaws; and Colubridae used their rear-positioned fangs by alternate jaw movement to damage the prey's surface.

## Teaser

**Highlighted Article:** Comparison of prey strikes of 36 venomous snake species using high-speed 3D kinematical analysis shows vipers are often faster than elapid snakes, with highest strike velocities in ambush predators, predators of mammals and larger snakes.

## INTRODUCTION

Few actions in nature inspire more fear and fascination than the strike of a venomous snake. Within half a second, snakes can start from a static ambush position, lunge at their prey, make contact and return to a resting position (Janoo and Gasc, 1992); in that same time, they must also erect their fangs, penetrate the prey and inject a dose of venom (LaDuc, 2002; Kardong and Bels, 1998). The high velocities and accelerations used during these behaviours make it difficult to observe these movements with the naked eye. Lack of high-speed imaging greatly limited the study and accurate description of snake kinematics in the past (Van Riper, 1953; Lester, 1955). As technology has advanced, venomous snakes have become the subject of many biomechanical studies, capturing their very fast strikes that appeared to happen within the blink of an eye. There was, however, a bias in the species studied, often being oriented towards the most feared viperid genera such as *Crotalus*, *Agkistrodon*, *Vipera*, *Bitis* and *Bothrops* (Janoo and Gasc, 1992; LaDuc, 2002; Kardong and Bels, 1998; Araújo and Martins, 2007; Higham et al., 2017). This research led to the conclusion that viperid snake strikes are much faster than those of any other snake (Vincent et al., 2005; Young, 2010). In more recent years, researchers have contradicted this conclusion by capturing strikes of colubrids, cobras and pythons (Nasoori et al., 2016; Penning et al., 2016; Kardong, 1986).

The urge to capture snake strikes started in the early 1950s with the development of high-speed photography by means of the electronic flash. The goal was to capture the snake at the moment it hits the prey in order to investigate whether they only stab their fangs into a prey or truly bite using their jaws (Van Riper, 1953). Using this method, only one picture per strike was captured. The authors were also handicapped because human reflexes are too slow to reliably set off the flash at the right moment. Fortunately, in the mid 1950s, high-speed motion pictures became available and were used to capture the first snake strike on film (Lester, 1955). Since that time, high-speed videography has improved dramatically. However, in most of the recent studies, this rapid motion has only been recorded using one camera, and often using relatively low resolution (Araújo and Martins, 2007; Kardong, 1986; Webb and Shine, 1998; Alfaro, 2002; Clark, 2006; Glaudas and Winne, 2007; Clark et al., 2012). These low resolution recordings are often seen in field studies where camera equipment and low lighting conditions limit the ability to reach higher frames per second.

Another common theme in studies on snake strike kinematics is that they often focus on a single snake species (Janoo and Gasc, 1992; LaDuc, 2002; Kardong and Bels, 1998; Young, 2010; Ryerson and Tan, 2017; Webb and Shine, 1998; Clark, 2006; Glaudas and Winne, 2007; Herrel et al., 2011; Penning et al., 2020) or a limited number of species belonging to a single genus (Araújo and Martins, 2007; Alfaro, 2002; Clark et al., 2012; Young et al., 2001; Alfaro, 2003; Whitford et al., 2020). One exception to this pattern, however, is a study by Cundall (2009) which examined 86 viperid species across 31 genera, investigating the occurrence of fang repositioning after prey contact at either 60 or 250 frames s^−1^. Comparative behavioural studies of feeding strikes across venomous snake families are also still rare, with the exception of a study comparing strike performance between the Texas ratsnake (colubrid) and two viperid species (Penning et al., 2016).

Previous studies have divided the strike into four distinct stages. (1) The extension stage is where the anterior one-third of the snake's body straightens from a curled position. (2) In the contact stage, the jaws hit the prey. The mouth is then closed and the neck is arched forward (around 80 ms in rattlesnake). (3) During the release stage, the jaws open and the fangs are extracted (around 120 ms in rattlesnakes). (4) The snake returns to a normal curled resting position during the retraction stage (between 130 and 160 ms in rattlesnakes).

Within venomous snakes there also are clear kinematic differences between defensive and predatory strikes. The attack strategy thus varies depending on whether they are lunging at their prey or at a potential threat. In predatory bites, the jaws open less wide and the mandible often makes first contact, followed by penetration of the fangs and venom injection (Hayes et al., 2002; Moon et al., 2019). When scaring off potential aggressors, snakes open their mouths very wide while lunging towards the aggressor, which is not always followed by a bite (Moon et al., 2019). When a bite occurs, it is most often without venom injection and is referred to as a dry bite (Cundall and Greene, 2000). Apart from the difference in gape, defensive strikes have been found to reach higher velocities (LaDuc, 2002). However, this may be a consequence of snakes striking at aggressors from further away, resulting in higher velocities when striking with a constant acceleration (LaDuc, 2002). Other studies have focused on the effect of the surroundings and body temperature on strike kinematics. Increased temperature was found to positively affect strike performance in rattlesnakes, with higher strike probability, velocity and angular gape velocity (Whitford et al., 2020). Recent studies have investigated the role of ontogeny on strike performance, where findings suggest juveniles can reach comparable velocities, although they strike over shorter distances (Ryerson and Tan, 2017). The latest findings on juvenile performance even suggest they are faster than their older conspecifics; the reason for a decline in performance with age remains unclear (Ryerson, 2020).

The drastic improvement in high-speed video cameras (1000 frames s^−1^ or higher) and the improvement of computer software that can combine 2D coordinates from different cameras into a 3D trajectory (Higham et al., 2017) makes this the ideal time to compare strike performance across a large number of venomous snake species and families. The use of 3D tracking reduces the error in interpreting movement from a single view, and enables more accurate reconstruction of 3D kinematics.

In this study, we examined the largest number of venomous snake species so far within a common-garden experimental set-up. This allowed us to perform a comparative analysis of 3D strike performance across 36 venomous snake species that span 23 genera and three families (Viperidae, Elapidae, Colubridae; Table 1). We investigated the variation in strike kinematics in these three families, including head movement, jaw opening and fang erection. We determined how Viperidae differ in their peak velocity and whether any of the Elapidae tested can reach comparable velocities and accelerations.

**Table 1.** Mean (±s.e.m.) kinematic values for three strikes for each individual snake across three families: Viperidae, Elapidae and Colubridae

Species	Peak *V* (m s^−1^)	Time to max. *V* (ms)	Peak *A* (m s^−2^)	GA (deg)	GV (deg s^−1^)	Distance (mm)
Colubridae						
* Boiga dendrophila*	1.82±0.69	68.67±24.66	269.67±86.52	23.91±10.70	1.32±0.54	80.08±18.57
Elapidae						
* Acanthophis rugosus*	2.21±0.21	30.00±11.79	80.67±18.98	98.88±50.52	11.19±7.23	95.97±10.18
* Aspidelaps lubricus*	0.32±0.05	142.67±58.62	18.67±3.53	13.96±2.40	1.16±0.50	58.25±5.53
* Naja melanoleuca*	1.00±0.21	73.33±8.29	58.33±6.17	72.68±21.01	4.22±1.53	57.90±5.11
* Walterinnesia aegyptia*	0.98±0.10	342.33±202.38	48.03±15.45	102.41±34.30	15.83±12.72	59.95±14.63
Viperidae						
* Agkistrodon contortrix*	2.84±0.08	47.33±6.64	331.67±113.39	38.88±16.18	2.99±0.73	101.85±7.44
* Agkistrodon taylori*	2.69±0.31	52.33±7.45	135.67±25.67	71.25±7.05	4.24±1.05	95.35±21.94
* Atropoides mexicanus*	2.08±0.23	78.00±10.69	167.33±22.78	90.08±24.35	4.42±1.01	101.88±16.38
* Bitis nasicornis*	3.21±0.49	42.67±5.61	247.33±72.63	95.67±29.03	14.52±3.09	61.16±5.12
* Bothriechis schlegelii*	1.56±0.06	64.00±19.61	84.00±12.01	96.32±28.43	6.51±2.81	71.26±8.83
* Bothrops asper*	3.53±0.98	117.33±30.07	276.33±78.89	152.73±26.62	8.37±2.86	157.09±69.54
* Bothrops atrox*	3.14±0.10	57.67±23.07	130.67±21.23	67.28±24.26	11.04±2.21	73.75±5.41
* Bothrops taeniatus*	2.42±0.07	58.50±41.50	296.00±41.68	90.37±34.76	15.33±6.39	92.89±9.81
* Cerastes cerastes*	2.58±0.30	36.33±5.24	76.33±11.55	78.81±18.15	9.43±4.16	86.62±7.10
* Crotalus atrox*	2.81±0.48	75.67±19.65	142.67±26.61	144.41±24.92	16.06±9.68	69.62±5.42
* Crotalus lepidus*	2.47±0.19	43.33±4.91	115.67±8.95	96.13±35.15	7.41±3.38	71.03±1.02
* Crotalus oreganus*	2.56±0.17	34.67±8.57	222.00±116.12	92.61±25.43	9.73±6.42	80.16±9.00
* Crotalus scutulatus*	2.45±0.39	208.33±80.11	120.00±48.17	113.74±22.76	9.41±2.99	63.31±9.57
* Daboia palaestinae*	1.81±0.15	35.00±7.51	75.33±14.25	49.12±36.10	4.22±2.90	72.98±13.42
* Deinagkistrodon acutus*	2.62±0.16	68.33±20.54	189.00±24.01	104.77±32.07	7.92±0.99	87.34±9.52
* Echis carinatus*	2.06±0.22	42.33±5.21	104.67±13.35	109.01±44.89	18.03±8.49	114.92±18.20
* Echis leucogaster*	1.56±0.51	66.00±13.43	141.67±44.85	143.11±4.25	8.71±1.12	99.44±22.18
* Echis ocellatus*	0.93±0.20	64.33±13.04	65.33±11.26	27.73±13.69	2.79±1.86	60.79±7.71
* Eristicophis macmahoni*	2.46±0.19	38.33±11.10	111.67±25.30	101.62±38.45	15.09±6.18	73.37±9.63
* Hypnale hypnale*	1.37±0.07	31.33±5.24	73.33±8.88	34.28±16.68	1.52±0.33	64.43±7.14
* Macrovipera lebetina*	3.37±0.09	21.67±2.91	298.33±205.89	100.11±26.32	25.31±19.63	105.54±1.34
* Porthidium ophryomegas*	1.66±0.38	68.67±5.36	113.00±12.50	89.33±40.03	15.16±8.68	71.30±8.33
* Proatheris superciliaris*	1.17±0.23	90.67±4.91	69.00±7.09	119.19±25.62	4.02±1.20	105.46±18.35
* Protobothrops cornutus*	3.50±0	45.67±0.88	180.33±40.76	119.34±32.53	19.60±10.44	108.78±12.63
* Protobothrops jerdonii*	2.30±0.30	70.00±5.51	119.00±9.24	153.57±21.98	11.15±4.30	86.63±5.25
* Protobothrops mucrosquamatus*	1.99±0.35	120.33±26.03	116.33±25.96	150.36±18.72	22.63±18.20	125.56±23.69
* Trimeresurus albolabris*	1.87±0.26	99.67±22.04	82.00±9.54	87.70±14.48	4.91±1.11	82.72±15.19
* Trimeresurus trigonocephalus*	1.85±0.20	71.67±11.84	140.00±4.16	94.11±27.47	8.72±3.55	106.33±17.30
* Vipera ammodytes*	2.01±0.38	49.67±5.21	129.67±19.81	95.53±6.59	10.60±3.35	73.88±13.93
* Vipera aspis*	2.29±0.49	35.67±13.17	181.33±34.35	113.89±32.61	4.46±2.06	100.26±3.72
* Vipera latastei*	2.59±0.18	39.33±6.89	133.33±15.50	115.33±10.52	14.35±8.26	76.02±6.70

*V*, velocity; *A*, acceleration; GA, gape angle; GV, gape velocity.

We tested whether ecological factors affect the kinematical performance in Viperidae, including predation strategy, diet, habitat, habitat temperature and biorhythm; additionally, the effect of distance to prey, snake head size and the part of the jaw that makes first contact with prey were investigated. We hypothesised that larger snakes reach higher velocities (DeVries et al., 2012) and accelerations, that the distance to the prey at the start of a strike is correlated with higher velocities (Vincent et al., 2005), that snakes closer to prey will open their jaws faster, and that strike kinematics will affect which part of the jaw first contacts prey.

## MATERIALS AND METHODS

### Animals and husbandry

**Fig. 1.**
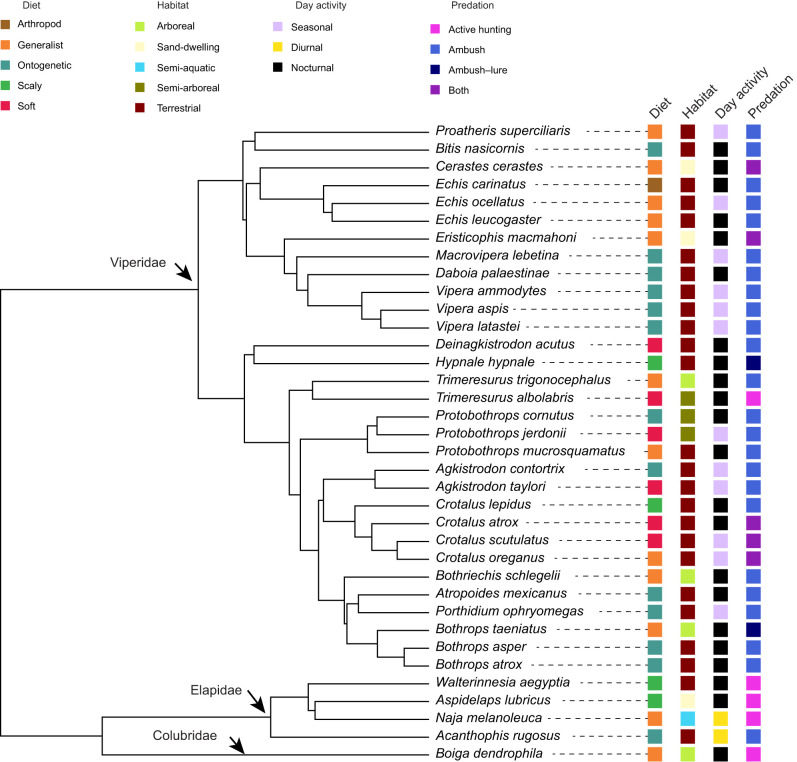
**Snake species included in this study and the distribution of their ecological factors.** Time-scaled phylogeny from Title et al. (2024). Assignments for diet, habitat, predation style and biorhythm were sourced from the literature (see Materials and Methods and Table S1).

Strikes were recorded for 36 species of venomous snakes (31 Viperidae, 4 Elapidae and 1 Colubridae) across 23 genera (18 Viperidae, 4 Elapidae and 1 Colubridae) (Fig. 1; Table S1). For each species, one individual was included in this study. One additional colubrid species, *Toxicodryas pulverulenta*, was included in the behavioural analyses only as we were unable to gather enough kinematic data for it. All snakes were housed at Venomworld, a venom production institution in Paris, France. Snakes were habituated to regular handling, but did not show any signs of tameness due to captivity. They were housed separately in clear containers in specialised reptile shelving units in a room that has skylights to provide a natural day/night cycle for the snakes. The mean room temperature varies from 18°C to 28°C from summer to winter to allow brumation. Access to the room was limited to the two snake handlers that own the company and authorised personnel familiar with the experimental and safety procedures. Institutional and national guidelines for the care and use of animals were followed and all experimental procedures involving animals were approved by the Biological Sciences Animal Ethics Committee (BSCI AEC), Monash University, VIC, Australia (project number 18428). The diet, habitat, mean habitat temperature, predation strategy and biorhythm for each species were collated from the literature (Fig. 1; Table S1) (Higham et al., 2017; Herrel et al., 2011; Shine, 1991; Shine et al., 2014; Wickum et al., 2007; Luiselli and Angelici, 2000; Calvete et al., 2021; Sutton et al., 2017; Porras et al., 2013; Hampton, 2011; Luiselli, 2006; Lomonte et al., 2008; Wasko and Sasa, 2009; Martins et al., 2001; Al-Sadoon, 1991; Beaupre, 1996; Putman and Clark, 2017; Martínez-Freiría et al., 2017; Lee, 2005; Richards et al., 2012; Emmanuel, 2005; Sawant et al., 2010; Jestrzemski and Kuzyakova, 2019; Leenders, 2019; Youngman et al., 2021; Luu et al., 2015; Guo and Zhao, 2006; Muntean et al., 2009; Naulleau and Bonnet, 1995; Santos et al., 2007; Oliveira and Martins, 2001).

### Strike target

The object used in our experiments was made of ballistic/medical gel which resembles the structure of skin and muscle (Gelatine #2, Humimic Medical). The ballistic gel was cut in small pieces, melted at 120°C for 30 min and cast, resulting in cylinders with a length of 9.5 cm and a diameter of 3.7 cm. This cylinder was warmed to 35–37°C to mimic body temperature, two dots were added to resemble eyes and it was mounted on a snake handling hook in order to introduce it into the recording arena. The gels were removed and replaced after each successful strike. The used gels were re-melted and re-cast at the end of each filming day.

### Experimental set-up

**Fig. 2.**
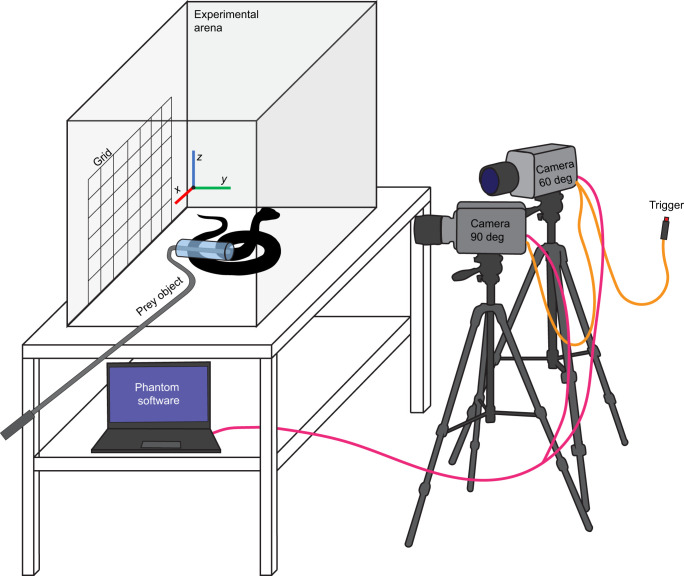
**Common garden equipment setup for snake strikes.** The experimental arena (140×70×70 cm) contained fresh cardboard flooring and was blinded on the back and right-side panel to minimise outside distractions. Two high-speed Phantom Miro M110 cameras were placed at 90 and 60 deg angles to the direction of the snake strike. The cameras continually recorded and temporarily stored the video footage on a flash drive; after the snake finished its strike, the cameras were triggered (orange cable) and the previous 4000 frames were stored from the flash drive to the computer (pink cable). Workflow of experiments: (1) snake transferred from regular housing to experimental arena; (2) 5 min acclimation; (3) introduction of prey object mounted on snake handling hook; (4) snake strikes; (5) trigger pushed; (6) recording checked on computer; (7) previous steps repeated until three successful strikes are recorded or the snake shows no interest in the prey item.

For each snake, between three and eight strikes were recorded in lateral view (90 deg) and slightly angled view (60 deg) using two Phantom Miro M110 cameras (Vision Research, Wayne, NJ, USA) with Nikon Nikkor AF 50 mm lenses. Strikes were recorded at 1000 frames s^−1^ with a throughput of 1.6 Gpx s^−1^ (Fig. 2). A total of 108 successful strike videos were recorded during May 2019. This resulted in three successful strike videos for each of the 36 species included (Table S2). At the start of each filming day and every time the cameras were moved, the ProAnalyst^®^ 6 inch calibration grid was recorded. Lighting was placed parallel to the 90 deg camera and angled down at 45 deg, and an additional LED spotlight was placed on top of the arena. A snake was transferred by snake hook to the 140×70×70 cm recording arena (length×width×height) lined with a fresh cardboard flooring. The prey object was introduced when the snake stopped investigating and settled down into a curled resting position (Fig. 2). When necessary, a snake hook was used to reposition the snake to the back of the recording arena before prey introduction. The ballistic gel was brought in front of the snake and was repeatedly pulled back quickly and brought closer again until a bite occurred or until 10 min elapsed. One of the experimenters was holding the hook with the ballistic gel attached when the snake bit the gel. This technique proved successful in most snakes; for some snake species, it was necessary to tap their tail to initiate a strike. This was repeated until three successful strikes were recorded of the same individual or until the snake stopped showing interest in the gel and/or tried to hide. When this occurred, the individual was returned to its enclosure, and reintroduced into the arena the next filming day. When the snake also did not show any interest in interacting with the prey object the following day, another individual from that species was used. Every time this occurred, we were able to get three successful strikes from the second individual. Where more than three videos were recorded for an individual, we selected the three trials with the best visibility of the morphological landmarks. A list of the three videos that were included for the kinematical analysis of each individual is given in Table S1. The Phantom Miro M110 cameras were triggered at the end of each strike and the last 4000 frames were saved from the internal flash memory.

### Video analyses

#### Tracking video data

**Fig. 3.**
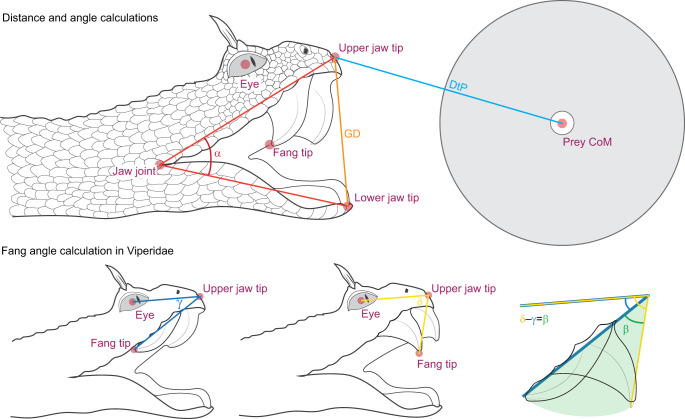
**Calculation of kinematic angles for head and fang.** Six landmarks were placed on every frame. Landmarks are shown as red dots: eye, upper jaw tip, jaw joint, lower jaw tip, fang tip and prey centre of mass (CoM). The distance between the upper jaw tip and lower jaw tip is the gape distance (GD), while the angle between the upper jaw tip, jaw point and lower jaw tip is the gape angle (α). The distance to prey (DtP) is calculated using the upper jaw tip and prey CoM. In viperid species, the fang angle is also calculated. The difference in angle between the eye, upper jaw tip and fang tip is used to calculate the angular velocity of fang erection. To get an estimation of the maximum angle of fang extension (β), the minimum angle (γ) between the eye, upper jaw tip and fang tip is subtracted from the maximum angle (δ).

The recorded videos were processed using ProAnalyst^®^ 3D software (Xcitex Inc., Cambridge, MA, USA) resulting in 3D coordinates of the snake's strike trajectory. Using the calibration grid and file (6 inch calibration cube with a grid of landmarks positioned 15 mm apart, 3DP-20170912-6.fixt) the ProAnalyst software calibrated the distance and stitched the landmarks of the two camera views together to create three-dimensional coordinates of the strike path, where position could be determined to the nearest 0.25 mm. Six landmarks were traced on both camera views at each time point (1000 frames s^−1^): (1) centre of the eye; (2) tip of the lower jaw; (3) tip of the upper jaw; (4) mouth corner; (5) tip of the fang; (6) centre of the prey object (Fig. 3). Both the 2D and 3D coordinates were exported to Microsoft Excel. Movie 1 shows the procedure for combining two views into 3D tracking of landmarks.

#### Kinematic analysis

The 3D coordinates were used to extract: (1) duration of strike (from first head movement to prey collision); (2) distance to prey (distance from the upper jaw to the prey at the start of the strike); (3) velocity of strike; (4) acceleration of strike; (5) maximum gape angle; (6) gape distance; (7) angular velocity and (8) acceleration of jaw opening; (9) fang angle (only in Viperidae); and (10) angular velocity and acceleration of fang (only for Viperidae), using R Statistical Environment v4.4.2 (http://www.R-project.org/) and RStudio (v2024.09.1; https://posit.co/download/rstudio-desktop/).

#### On-screen measurements

Video frames were imported into ImageJ to measure the total head length of each individual. For the Viperidae, the angle of first fang prey contact was also measured.

#### Behavioural notes

**Fig. 4.**
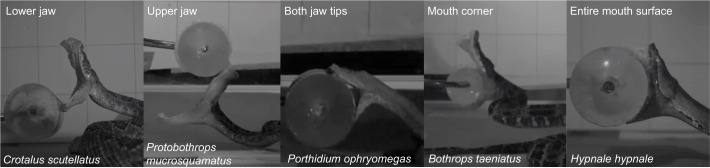
**The part of the jaw that makes first contact with the prey varies.** Images of snakes at the moment of first prey contact. Examples for each contact mode are given: lower jaw, upper jaw, both jaw tips, mouth corner and entire surface of the mouth. Some snakes did not consistently show the same prey contact and were categorised as ‘varied’ (V).

For each video, the type of bite was placed into 6 categories (Fig. 4) according to which part of the snake touched the prey item first: lower jaw first (‘MAN’ strike; Cundall and Greene, 2000; categorised as L); upper jaw first (‘PMX’ strike, U); corner of mouth first after which the jaws make contact as a result of mouth closing (M); entire mouth surface (F); both jaw tips at the same time (J); or it varied across the three strikes recorded (V).

### Kinematic and statistical analyses

The 3D coordinates of the strike path for each landmark were analysed in R (v3.4.3) and RStudio (v1.70). All coordinates were smoothed using a cubic spline (smooth.spline from the stats package in R) with smoothing parameter 0.5 for *x* and *z* coordinates and 0.75 for *y* coordinates [as accuracy in positioning in the *y* axis (the most parallel to the camera views) was lower, resulting in higher noise]. From the smoothed coordinates, the following parameters were calculated: peak velocity and acceleration (eye path); distance to prey (Fig. 3, DtP); gape distance (Fig. 3, GD); gape angle (Fig. 3, α); and angular velocity and acceleration of mouth opening. For Viperidae only, two additional parameters were included: angle at full fang erection (Fig. 3, β) and the angular velocity and acceleration with which the fang erects. The angle of fang erection was calculated by subtracting the minimum angle (γ) from the maximum angle (δ) between fang tip, upper jaw and eye (Fig. 3). Velocities were calculated as the first-order derivative of displacement (Eqn 1) and accelerations as the second-order derivative of displacement (Eqn 2) (Higham et al., 2017; Penning et al., 2016, 2020; Anderson et al., 2016; Herrel et al., 2011):
(1)

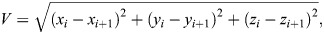

(2)

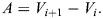
An example of smoothed displacement, velocity and acceleration is shown in Fig. S1. Differences in kinematical values against ecological factors (diet, predation style, biorhythm, habitat type and habitat temperature) were tested using the R package phylolm (Ho and Ane, 2014). The diet categories we assigned were arthropod, scaly (vertebrate prey), soft (vertebrate prey), generalist (no dietary pattern) and ontogenetic (diet varies with life stage). For habitat, the categories were semi-aquatic, sand-dwelling, arboreal (spending substantial time in trees), semi-arboreal and terrestrial (spending majority of time at ground level). Biorhythm was determined by activity patterns, assigned to nocturnal (active at night), diurnal (active during the day) and seasonal (variable activity pattern throughout the year). Predation category was assigned as ambush (lie-and-wait ambush strategy), ambush–lure (ambush strategy using a tail lure), active (pursuit) or both (variety of predation strategies).

For each test, a species mean of one kinematic variable was tested against one predictor at a time (diet, habitat, habitat temperature, day activity, predation, head size or jaw hitting). We also tested head size against the other predictor variables, and start distance against the other kinematic variables. Each phylolm test included a phylogenetic signal test measuring lambda. We used the ultrametric constrained tree (Title et al., 2024) for all tests. When important differences were found, they were visualised in scatterplots and violin plots using *ggplot2* (Wickham, 2016). To investigate the relationship between certain parameters, least squares regressions of log-transformed parameters were plotted using *ggplot2*.

Strike velocities across the different trials within individuals were compared in order to check whether these varied, particularly to see whether snakes tired out and performed less well in their second and third strike. The difference in peak velocity for strike A, B and C was calculated for each individual (A–B, A–C and B–C), and used to obtain the percentage of positive and negative changes in velocity and to calculate the mean difference in velocity for consecutive strikes. To visualise the difference in strike velocity across the three strikes, A was subtracted from all values so that A=0, B−A and C−A, which were then plotted using *ggplot2* (Wickham, 2016).

## RESULTS

Strike kinematics for all 36 species included rapid acceleration of the anterior portion of the body with almost simultaneous mouth opening.

Overall, we found viperids to have rapid strikes with a smooth penetration of the fangs into the gel. In 50% of the viperid species, one or both of the fangs were removed from the gel and repenetrated at a more favourable angle. This only appeared to occur when the initial penetration was not favourable. When the bite location was acceptable but the fang was only slightly misplaced, the fang was repositioned very close to the original penetration site. When the snake only just reached its prey and needed a better grip, the fang was removed and that side of the jaw was moved forward, ‘walking’ the fang further onto the prey so that it could be repenetrated in a better position. In this situation, fang walking was often followed by the repositioning of the other fang. When the prey was released, the fangs were often observed to fold back one at a time rather than simultaneously.

**Fig. 5.**
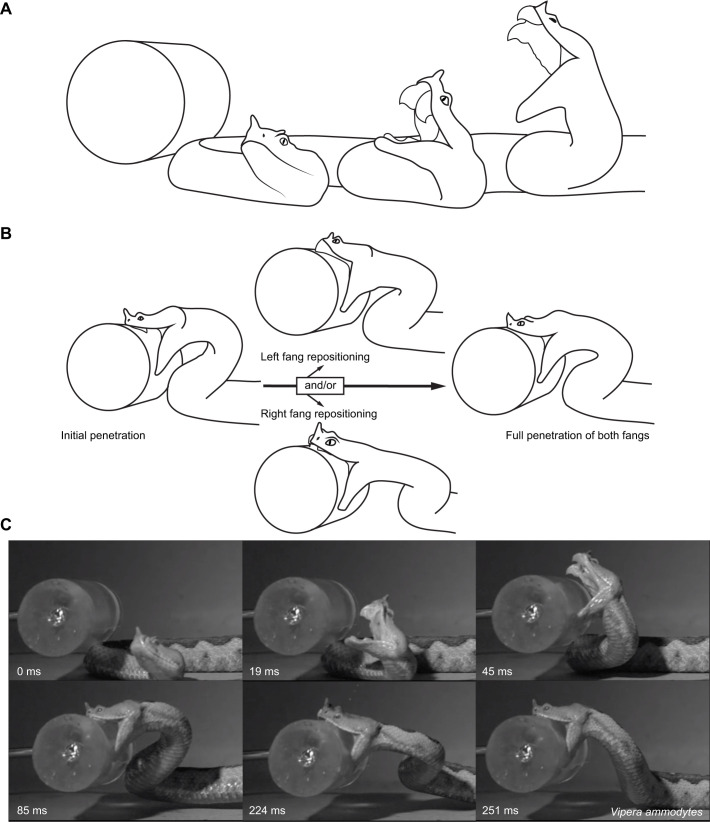
**Outline drawings and frames of the strike of *Vipera ammodytes* show a typical strike sequence for vipers.** (A) Strike – head movement and gape expansion. The initial lunge made at the prey is followed by fang penetration. (B) Prey manipulation – fang repositioning. When the initial penetration does not allow good venom injection, one or both of the fangs are repositioned. (C) Corresponding video frames showing the timing of the strike sequence (Movie 2).

A typical viperid strike cycle is represented by outline drawings based on video frames of *Vipera ammodytes* (Fig. 5; Movie 2). This shows the lunge (Fig. 5A) as well as fang repositioning in viperid species (Fig. 5B). The corresponding video frames are shown with the time in milliseconds (Fig. 5C). In Viperidae, as soon as the snake starts its explosive lunge by the extension of its curved neck and trunk, the jaw starts to open, reaching the largest gape very shortly before the prey is reached. Only after first prey contact is made do the jaws close around the prey. Venom was usually injected when the fang was fully embedded in the prey. In the videos, the injection of venom can be verified by venom shooting or gushing out of the gel during injection or at prey release. In a very few cases, the fang started leaking venom before penetration (*Bitis nasicornis*, Movie 3). This, however, could be the result of over-productive venom glands due to the frequent milking of the individuals used in this study. Venom injection could also be identified visually by a cloud that appears in the gel, but was not always noticeable in the videos. Finally, venom injection could be confirmed when droplets of venom were present on the gel when it was removed from the experimental arena (Fig. S2A).

The Elapidae showed a different strike pattern. The snakes often crept closer to the prey first, and when they were close enough, they lunged and opened their mouth. However, this lunge was less explosive than that of viperids, and they did not start from a completely curled position. After the first bite, they slightly released and bit again a few times. This repeated biting action is likely a result of the muscles tensing repeatedly to squeeze the venom gland and inject more venom (see *Aspidelaps lubricus*, Movie 4).

The Colubridae included in this study are rear-fanged and were found to have a very different strike. They reached maximum gape sooner and often lunged across greater distances at this maximum gape. After prey contact, they closed their mouths and started to alternatingly drag their maxilla across the prey surface, resulting in two crescent-shaped cuts (Fig. S2B). This large wound likely ensures good venom transfer into the prey. This was best observed in video 2A of *Toxicodryas pulverulenta* (Movie 5; this individual was not included in the kinematical analysis).

We observed some strikes where the snakes seemed unable to predict the distance of the prey item, resulting in them barely reaching their prey, after which vipers repositioned their fangs one or more times to achieve a better grip. At other times, the snake reached the prey sooner than expected. For example, *Macrovipera lebetina* hit its right fang and broke it off (*Macrovipera lebetina*, Movie 6). This is likely how fang loss during feeding most often occurs, but this is the first time it has been caught on camera. Fangs have previously been found in snake scats, which means fangs often break off during prey penetration, remain stuck in the prey and are swallowed by the snake; during our experiments, no fangs were left behind in the gel.

**Fig. 6.**
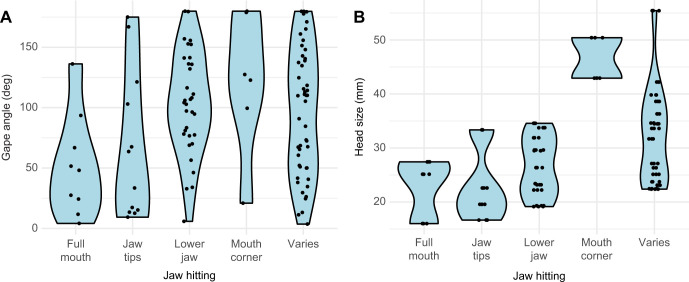
**The area of the jaw that first contacts prey varies with gape angle and head size.** Differences in the part of the mouth that makes first contact across gape angle (A) and head size (B). Each dot on the plots represents an individual trial (three trials per individual).

The biggest variable in the strike pattern across all snakes was which part of the jaw made first contact with the prey. No clear pattern was observed within individuals, as the point that makes first contact varied across strikes (Table S3). For phylolm analyses of vipers, we did find, however, that the part of the jaw that first makes contact with prey differed significantly with maximum gape angle (lambda=0, adjusted *R*^2^=0.17) as large gapes result in either the mouth corner making first contact (slope=70.0, s.e.=27.2, *t*=2.6, *P*=0.02) or the lower jaw (slope=56.7, s.e.=20.1, *t*=2.8, *P*=0.01), while smaller gapes most often result in the entire surface of the mouth making first contact (slope=51.5, s.e.=17.2, *t*=3, *P*=0.01) (Fig. 6A; Table S3). Having said this, it is important to note that mouth corner contact is also correlated with large head size in vipers (lambda=0, adjusted *R*^2^=0.31, slope=23.8, s.e.=7.0, *t*=3.4, *P*<0.01) (Fig. 6B; Table S4).

### Kinematic analysis

All kinematic values for each of the 36 species are shown in Table 1. In most Viperidae, the prey was reached within the first 100 ms, with a maximum of 208.3±80.1 ms (mean±s.e.m.; *Crotalus scutulatus*) and a minimum of 21.7±2.9 ms (*Macrovipera lebetina*) (Table 1). Elapidae showed a wider span, with *Acantophis rugosus* and *Naja melanoleuca* reaching their prey equally fast as most viperids (30.0±11.8 and 73.3±8.3 ms, respectively), while *Walterinnesia aegyptia* took much longer (342.3±202.4 ms).

**Fig. 7.**
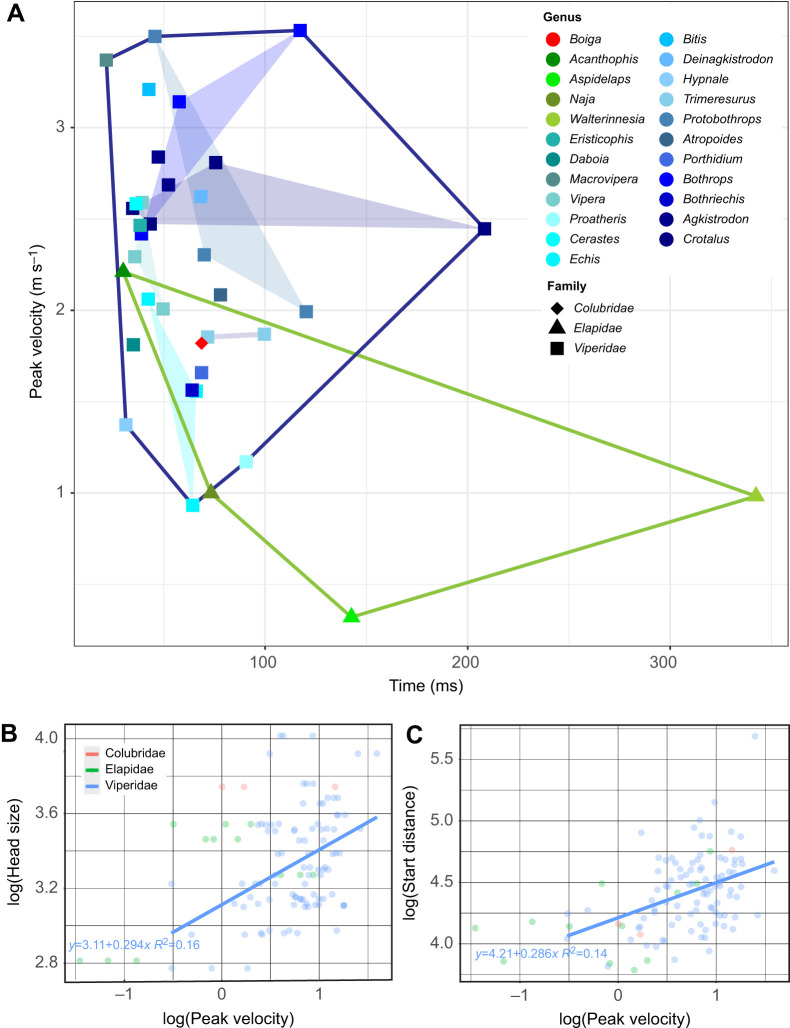
**Peak velocity of each snake strike varies with head size and distance from prey.** (A) Scatterplot of peak velocity against the time it was reached. Shapes and clear hulls show family, colours and filled hulls show genus. (B) Regression of head size against peak velocity. (C) Regression of distance to prey against peak velocity. In all panels, family is also shown by colour: Colubridae, red; Elapidae, green tones; and Viperidae, blue tones. Each dot on the regression plots represents an individual trial (three trials per individual).

The peak velocity of many viperid species was higher than those observed in the elapid and colubrid species included in this study (Fig. 7A). *Bothrops asper* reached the highest peak velocity in viperids (3.53±0.98 m s^−1^) and *Acanthophis rugosus* in elapids (2.21±0.21 m s^−1^), while the slowest members of each family were *Echis ocellatus* and *Aspidelaps lubricus* (0.93±0.20 m s^−1^ and 0.32±0.05 m s^−1^, respectively).

Only vipers had an adequate sample for the phylolm statistical tests presented below. A positive correlation between head size was found with both peak velocity (slope=0.03, s.e.=0.01, *t*=2.5, *P*=0.02) and acceleration (slope=3.6, s.e.=1.3, *t*=2.7, *P*=0.01) (Fig. 7B; Table S3), so the larger the snake, the higher the acceleration and the higher the velocity reached. Distance at the start of the strike also had a significant correlation with peak acceleration (slope=1.3, s.e.=0.6, *t*=2.3, *P*=0.03), gape angle (slope=0.7, s.e.=0.3, *t*=2.8, *P*=0.01) and contact angle (slope=0.6, s.e.=0.04, *t*=15.6, *P*<0.01) (Table S5). Several kinematic variables (peak velocity, peak acceleration, gape angle, start distance, contact angle) had significant differences depending on the part of the jaw that first made contact with the prey (see Table S3).

**Fig. 8.**
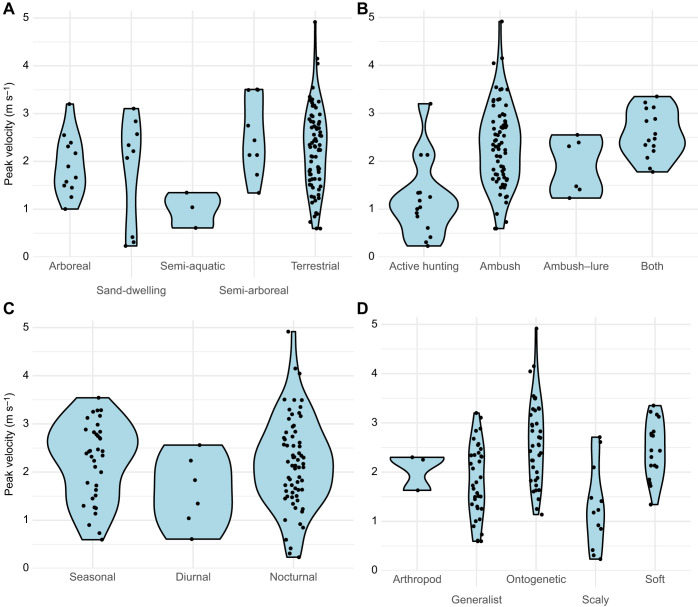
**Peak velocity during strikes is not correlated with ecological factors.** Variation in peak velocity for all snake strikes across (A) habitat, (B) predation, (C) activity pattern and (D) diet. Each dot on the plots represents an individual trial (three trials per individual).

Across the ecological factors (diet, predation style, biorhythm, habitat and environmental temperature), few significant differences were found. Semi-aquatic snakes appear to have the lowest peak velocities compared with semi-arboreal and terrestrial ones (Fig. 8). No difference in velocity was found across snakes that differed in biorhythm. For vipers, there was no significant relationship between peak velocity and diet or acceleration and diet (Table S3). There was a significant association between contact angle and diet (Table S3).

We also observed no clear signs of fatigue in the strike velocity in consecutive strikes: the first recorded velocity of a particular individual was not always faster or slower than that in subsequent trials. In 47.22% of cases, the first recorded velocity was highest. The average difference between two consecutive strikes was 0.58 m s^−1^, which can be both slower or faster (with positive variation between 0.01 and 1.38 m s^−1^ and negative variation between 0.08 and 1.62 m s^−1^). Only one individual had a drastic decrease in velocity, *Bothrops asper*: its first strike had a peak velocity of 4.91 m s^−1^, second 4.05 m s^−1^ and third 1.63 m s^−1^. Similarly, only one individual had a drastic increase, *Boiga dendrophila*, with 1.00 m s^−1^ initially, followed by 1.25 m s^−1^ and a final velocity of 3.20 m s^−1^.

There was a wide variation in maximum gape angle, where on average the angle was around 93 deg. Not only did it vary greatly across species but also it varied within an individual. Large gapes were observed mostly in viperid genera such as *Vipera*, *Protobothrops*, *Echis* and some members from the genera *Bothrops* and *Crotalus*. The biggest viperid snake, *Atropoides mexicanus*, did not seem to reach its maximum gape; however, this could be due to the relatively small target size compared with its head size. Maximum gape was not affected by predation style (Table S3, Fig. S3B). For the distance to prey, there was a slight positive slope with maximum gape angle and gape angular velocity in viperids, so that viperids open their mouths wider when the prey is further away; however, the fit was very low (*R*^2^=0.041 and 0.017, respectively) (Fig. S3E,F). A slight negative slope was found in elapids, so they open their mouths wider and faster when the prey is closer (*R*^2^=0.02 and 0.021, respectively) (Fig. S3E,F).

In viperid species the fang erection was also tracked. Here, it was found that the fang was fully erected before the peak velocity of the strike was reached. Across the species, the maximum fang angle varied between 36 deg in *Macrovipera lebetina* and 162 deg in *Echis carinatus*, with an average maximum angle of 98 deg. No meaningful differences in fang angle or fang angle velocity were observed (Table S6). The angle of contact between the fang and the gel surface also varied greatly, with 58 deg in *Atropoides mexicanus* and 133 deg in *Echis carinatus*, with an average maximum angle of 99 deg. When regressed against head size, a negative correlation was found (lambda=0, adjusted *R*^2^=0.2, slope=−0.7, s.e.=0.3, *t*=−2.6, *P*<0.05), with large snakes having a smaller contact angle.

## DISCUSSION

Venomous snakes evolved quick strikes in order to capture their preferred prey. In mammals, the startle response can activate muscles in 14–151 ms, which results in actual movement within 60–395 ms (Davis, 1984; Yilmaz and Meister, 2013). Whether snakes can stay within the low end of this time was previously confirmed for three species, *Pantherophis obsoletus*, *Agkistrodon piscivorus* and *Crotalus atrox* (Penning et al., 2016). Our results further confirm this as 84% of the viperids included in this study reached their prey in under 90 ms and 55% within the first 60 ms, making them faster than the average mammalian response. When prey, however, are able to escape in time, snakes can still catch up as long as their acceleration is higher than the prey's flight acceleration. Jackrabbits (Carrier, 1995), for example, were found to have a jumping acceleration of 40 m s^−2^, which is lower than all the viperid peak accelerations recorded here, which range between 65 and 330 m s^−2^ (Table 1). Some prey, such as kangaroo rat, have flight accelerations of 950 m s^−2^ and thus far exceed snakes' abilities (Higham et al., 2017).

When comparing velocity across families, viperids were found to have higher velocities and accelerations on average, and consequently reached their prey faster. This difference in velocity could be partly explained by their preferred prey and predation strategy. Viperid snakes very often target mammalian prey with quick startle responses, using ambush hunting to do so. With this predation style, snakes initiate their strike from a static position and thus rely on their strike acceleration and accuracy to reach their prey in time (Webb and Shine, 1998). It has also been hypothesised that snakes that target smaller prey have higher acceleration because of the faster startle response in small prey species (Penning et al., 2016). We did not account for prey size, so further studies are needed to confirm this.

Those elapid species that target reptiles and use active pursuit to capture their prey took longer to reach their prey at lower peak velocities, as expected. However, there are also elapid species that specialise on mammalian prey and use ambush hunting. A good example is the rough-scaled death adder (*Acanthophis rugosus*), which resembles the viperid body shape with a more triangular head, and stouter and shorter body, and uses caudal tail luring. This resemblance to vipers is very striking and likely indicates potential convergent evolution of morphology, attack strategy and strike kinematics. When comparing the velocity and time until prey contact with those of vipers with similar lifestyles, they perform very similarly.

For colubrids, we were unfortunately only able to measure the kinematic parameters of one species, *Boiga dendrophila*, which reached a higher velocity than three out of the four elapids. In the literature, colubrid strike velocities have often been reported to be quite low (Greenwald, 1974; Alfaro, 2002; Bilcke et al., 2006); however, *Pantherophis obsoletus* has also been found to have a high strike velocity (Penning et al., 2016). Because of the inclusion of only one species of Colubridae, care has to be taken in generalising these results. Just as seen in viperids and elapids, colubrid species likely vary substantially in strike velocity and further research is needed on these rear-fanged snakes to gain a greater understanding of their performance.

Apart from kinematic differences across the families, we also found behavioural patterns. A typical viperid strike pattern is shown in Fig. 5, where they lunge at their prey from a still position, penetrate their long slender fangs at great velocity and have the ability to re-position them so they are fully penetrated for venom injection. Many elapids will stealthily creep forward to reduce the strike distance, after which they do a short lunge and bite; as soon as they have a tight grip, they slightly loosen their jaws and bite again repeatedly, likely to prolong the venom flow into their prey. Some elapids, however, resemble the viperid strike more, such as *Acanthophis rugosus* discussed above. Colubrids seem to use their rear-positioned fangs to create large wounds by dragging them across the prey's integument one side at a time, here observed in *Toxicodryas pulverulenta*. To our knowledge, this is the first time this behaviour has been filmed, although big gaping wounds are known to occur when prey are bitten by a colubrid snake.

When investigating the effect of head size as a proxy for snake size, a positive correlation was found with peak velocity and peak acceleration, with larger snakes having higher performance. A previous study on *Hoplocephalus bungaroides* found no effect of snout–vent length or mass on speed measures (Webb and Shine, 1998). That study investigated several individuals of one species, indicating body size has little effect within this species. The results presented here show for the first time that higher performance is correlated with snake size across a large number of species. Previously, this principle has been suggested when preliminary high-speed analyses on the heavy-bodied *Bitis arietans* showed that they are nearly twice as fast as previously recorded rattlesnakes (Young, 2010).

Distance to prey at the start of the strike also showed a positive correlation with peak velocity, such that the further away the prey is, the higher the velocity. This is the effect of the acceleration remaining positive, so that the velocity will keep increasing until the prey is reached or until the upper body is fully unfolded and the snake decelerates.

Maximum gape angle varied greatly within individuals and across species. Many snakes seem to be able to achieve very large gapes (>100 deg), but can only do so when the prey is at the ideal distance and when the approach towards the prey is optimal. If the prey is too close, snakes are unable to open their mouths fast enough and the maximum gape cannot be reached. When comparing angular velocity across ecological factors, no significant difference was found. To date, only one study has compared angular velocity across two *Crotalus* species and the authors did not detect any differences (Whitford et al., 2020). Larger snakes were found to have slightly larger gapes and achieved them faster. However, when looking at the largest snake species in this study, they had slightly less wide gapes. It is possible that target size also plays a role, where particularly large snakes will only open their mouths wide enough to easily get around the diameter of their target. Additional experiments varying the target size are needed.

A high variability in which part of the mouth contacts the prey first was also found. No clear pattern was detected within individuals or any of the ecological factors. However, it seems that maximum gape angle and head size likely play a role in this. Large heads and large gape angles result in snakes hitting their prey first with the mouth corner, after which the mouth is closed, resulting in the fangs being carefully embedded. Small gapes result in the entire mouth surface making contact, and in this case the fangs immediately embedded upon prey contact. The latter is often observed in snakes with small heads, but they are also observed to make first contact with both jaw tips or just the lower jaw. The size of the target likely plays an important role as well. In this experiment, the target may have been too big for the small snakes and possibly somewhat too small for the very big snakes. Future experiments should adjust the target size to a certain percentage of the snake head size or vary the target size to see what effect it has on the snake's bite pattern.

**Fig. 9.**
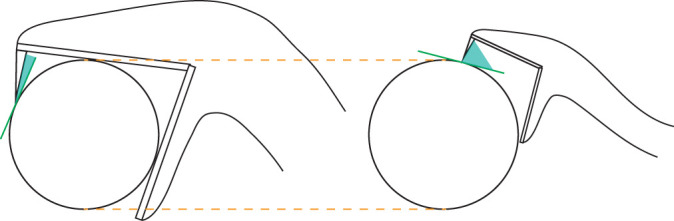
**Size discrepancy between a snake and its prey influences fang contact angle.** Constant prey size can lead to variation in fang contact angle when snakes vary in size.

Similarly, differences in the viperid fang contact angle were observed where head size was negatively correlated with contact angle. The fangs of large snakes are thus penetrated at a smaller angle to the prey's surface. This again seems to show an effect of the constant target size used across our experiments (Fig. 9). No differences were observed in fang angle or fang angular velocity across the ecological factors. We did find that the fang was fully erected before the prey was reached.

Kinematic values have previously been recorded for six species included in this study. The strike velocity of *Bitis nasicornis* and *Vipera ammodytes* were previously recorded by Janoo and Gasc (1992). Their values are lower than the ones recorded here with 0.39 m s^−1^ for *B. nasicornis* compared with our 3.21 m s^−1^, and 1.32 m s^−1^ for *V. ammodytes* compared with our 2.01 m s^−1^. This difference in velocity is likely due to the lower frame rate and likely also lower resolution of the video footage. Initially, the velocity of *Crotalus atrox* was estimated to be 2.61–3.71 m s^−1^ (LaDuc, 2002), and was narrowed down to 2.95 m s^−1^ (Penning et al., 2016), which corresponds very well with our 2.81 m s^−1^ for this species. *Trimeresurus albolabris* had a velocity between 1.45 and 1.6 m s^−1^ in an earlier study (Herrel et al., 2011); our calculation is only slightly higher with 1.87 m s^−1^. Lastly *Crotalus scutellatus* and *Crotalus oreganus* were included in a recent study looking at the effect of temperature on strike velocity, where temperature ranged from 15 to 35°C (Whitford et al., 2020). Their velocity values varied between 2.8 and 4.05 m s^−1^ for *C. scutellatus* and 3.23 and 4.06 m s^−1^ for *C. oreganus*, with velocity increasing with increasing temperature (Whitford et al., 2020). Our values are lower at 2.45 and 2.56 m s^−1^, respectively, which is likely a consequence of the lower ambient temperature of our experiments.

To our knowledge, the use of high-speed video analysis to study strike kinematics for vertebrates biting is largely restricted to snakes. Several studies have employed high-speed video analysis to study feeding kinematics on lizards (So et al., 1992; Wainwright et al., 1991; Herrel et al., 1996), frogs (Deban and Nishikawa, 1992), tadpoles (Venesky et al., 2011), fishes (Konow and Sanford, 2008), sharks (Ferry-Graham, 1998) and eels (Mehta and Wainwright, 2007). Of these, only the study on sharks specifically investigated biting prey. Kinematic analysis of feeding in lizards has largely focused on the high-speed prehensile tongues involved in prey capture. Likewise, high-speed video analysis of feeding in fishes and eels is largely concerned with suction feeding underwater. Therefore, there is potential to expand kinematic video analysis to investigate feeding in other tetrapods which otherwise would not have a high-speed component to their feeding. This would be particularly relevant to other tetrapods that employ ambush strategies when acquiring prey.

### Conclusion

This study is the largest of its kind to date. Many differences in strike performance were observed across the 36 species, with viperid snakes on average being faster. For colubrids, strike kinematics between the part of the jaw that first makes contact with the prey and start distance was correlated with peak acceleration, jaw gape angle and contact angle with prey. Additionally, clear behavioural differences have now been caught on camera at high frame rates, showing the fluent strike with the possibility of fang repositioning in viperids, the stealthy elapid strike with repetitive biting and the rear-fang slicing of colubrid snakes.

## Supplementary Material

10.1242/jexbio.250347_sup1 Supplementary information

Table S3. Results from phylolm analysis (Phylogenetic generalised least squares regression and phylogenetic signal) of kinematic variables against predictor variables for Viperidae. Significance indicated in bold.

Table S4. Results from phylolm analysis (Phylogenetic generalised least squares regression and phylogenetic signal) of head size against predictor variables for Viperidae. Significance indicated in bold.

Table S5. Results from phylolm analysis (Phylogenetic generalised least squares regression and phylogenetic signal) of Start Distance against kinematic variables for Viperidae. Significance indicated in bold.

Table S6. Results from phylolm analysis (Phylogenetic generalised least squares regression and phylogenetic signal) of fang kinematic variables against predictor variables for Viperidae. Significance indicated in bold.

## References

[JEB250347C1] Al-Sadoon, M. K. (1991). Metabolic rate-temperature curves of the horned viper, *Cerastes cerastes gasperetti*, the moila snake, *Malpolon moilensis*, and the adder, *Virera berus*. *Comp. Biochem. Physiol. A Physiol.* 99, 119-122. 10.1016/0300-9629(91)90245-8

[JEB250347C2] Alfaro, M. E. (2002). Forward attack modes of aquatic feeding garter snakes. *Funct. Ecol.* 16, 204-215. 10.1046/j.1365-2435.2002.00620.x

[JEB250347C3] Alfaro, M. E. (2003). Sweeping and striking: a kinematic study of the trunk during prey capture in three thamnophiine snakes. *J. Exp. Biol.* 206, 2381-2392. 10.1242/jeb.00424 12796455

[JEB250347C4] Anderson, P. S., Lacosse, J. and Pankow, M. (2016). Point of impact: the effect of size and speed on puncture mechanics. *Interface Focus* 6, 20150111. 10.1098/rsfs.2015.0111 27274801 PMC4843624

[JEB250347C5] Araújo, M. and Martins, M. (2007). The defensive strike of five species of lanceheads of the genus *Bothrops* (Viperidae). *Braz. J. Biol.* 67, 327-332. 10.1590/S1519-69842007000200019 17876444

[JEB250347C6] Beaupre, S. J. (1996). Field metabolic rate, water flux, and energy budgets of mottled rock rattlesnakes, *Crotalus lepidus*, from two populations. *Copeia* 1996, 319-329. 10.2307/1446847

[JEB250347C7] Bilcke, J., Herrel, A. and Van Damme, R. (2006). Correlated evolution of aquatic prey-capture strategies in European and American natricine snakes. *Biol. J. Linn. Soc.* 88, 73-83. 10.1111/j.1095-8312.2006.00608.x

[JEB250347C8] Calvete, J. J., Pla, D., Els, J., Carranza, S., Damm, M., Hempel, B.-F., John, E. B., Petras, D., Heiss, P. and Nalbantsoy, A. (2021). Combined molecular and elemental mass spectrometry approaches for absolute quantification of proteomes: application to the venomics characterization of the two species of desert black cobras, *Walterinnesia aegyptia* and *Walterinnesia morgani*. *J. Proteome Res.* 20, 5064-5078. 10.1021/acs.jproteome.1c00608 34606723 PMC8576837

[JEB250347C9] Carrier, D. R. (1995). Ontogeny of jumping performance in the black-tailed jackrabbit (*Lepus californicus*). *Zoology* 98, 309-309.

[JEB250347C10] Clark, R. W. (2006). Fixed videography to study predation behavior of an ambush foraging snake, *Crotalus horridus*. *Copeia* 2006, 181-187. 10.1643/0045-8511(2006)6[181:FVTSPB]2.0.CO;2

[JEB250347C11] Clark, R. W., Tangco, S. and Barbour, M. A. (2012). Field video recordings reveal factors influencing predatory strike success of free-ranging rattlesnakes (*Crotalus* spp.). *Anim. Behav.* 84, 183-190. 10.1016/j.anbehav.2012.04.029

[JEB250347C12] Cundall, D. (2009). Viper fangs: functional limitations of extreme teeth. *Physiol. Biochem. Zool.* 82, 63-79. 10.1086/594380 19025501

[JEB250347C13] Cundall, D. and Greene, H. (2000). Feeding in Snakes. In *Feeding: Form, Function, and Evolution in Tetrapod Vertebrates* (ed. K. Schwenk), pp. 293-333. Elsevier.

[JEB250347C14] Davis, M. (1984). *The Mammalian Startle Response: Neural Mechanisms of Startle Behavior*. Springer.

[JEB250347C15] Deban, S. M. and Nishikawa, K. C. (1992). The kinematics of prey capture and the mechanism of tongue protraction in the green tree frog *Hyla cinerea*. *J. Exp. Biol.* 170, 235-256. 10.1242/jeb.170.1.235

[JEB250347C16] Devries, M., Murphy, E. and Patek, S. (2012). Strike mechanics of an ambush predator: the spearing mantis shrimp. *J. Exp. Biol.* 215, 4374-4384. 10.1242/jeb.075317 23175528

[JEB250347C17] Emmanuel, B. (2005). Envenoming by the viperid snake *Eristicophis macmahonii*. *Toxicon* 46, 918-920. 10.1016/j.toxicon.2005.08.019 16269163

[JEB250347C18] Ferry-Graham, L. (1998). Feeding kinematics of hatchling swellsharks, *Cephaloscyllium ventriosum* (Scyliorhinidae): the importance of predator size. *Mar. Biol.* 131, 703-718. 10.1007/s002270050362

[JEB250347C19] Glaudas, X. and Winne, C. T. (2007). Do warning displays predict striking behavior in a viperid snake, the cottonmouth (*Agkistrodon piscivorus*)? *Can. J. Zool.* 85, 574-578. 10.1139/Z07-025

[JEB250347C20] Greenwald, O. (1974). Thermal dependence of striking and prey capture by gopher snakes. *Copeia* 1974, 141-148. 10.2307/1443016

[JEB250347C21] Guo, P. and Zhao, E.-M. (2006). Comparison of skull morphology in nine Asian pit vipers (Serpentes: Crotalinae). *Herpetol. J.* 16, 305-313.

[JEB250347C22] Hampton, P. M. (2011). Ventral and sub-caudal scale counts are associated with macrohabitat use and tail specialization in viperid snakes. *Evol. Ecol.* 25, 531-546. 10.1007/s10682-010-9432-z

[JEB250347C23] Hayes, W. K., Herbert, S. S., Rehling, G. C. and Gennaro, J. F. (2002). Factors that influence venom expenditure in viperids and other snake species during predatory and defensive contexts. In *Biology of the Vipers* (ed. G. W. H. Schuett, M. E. Matsdouglas, Greene and W. Harry). Eagle Mountain Pub Lc.

[JEB250347C24] Herrel, A., Cleuren, J. and Vree, F. D. (1996). Kinematics of feeding in the lizard *Agama stellio*. *J. Exp. Biol.* 199, 1727-1742. 10.1242/jeb.199.8.1727 9319635

[JEB250347C25] Herrel, A., Huyghe, K., Oković, P., Lisičić, D. and Tadić, Z. (2011). Fast and furious: effects of body size on strike performance in an arboreal viper *Trimeresurus* (*Cryptelytrops*) *albolabris*. *J. Exp. Zool. A Ecol. Genet. Physiol.* 315, 22-29. 10.1002/jez.645 20853417

[JEB250347C26] Higham, T. E., Clark, R. W., Collins, C. E., Whitford, M. D. and Freymiller, G. A. (2017). Rattlesnakes are extremely fast and variable when striking at kangaroo rats in nature: three-dimensional high-speed kinematics at night. *Sci. Rep.* 7, 40412. 10.1038/srep40412 28084400 PMC5234031

[JEB250347C27] Ho, L. S. T. and Ane, C. (2014). A linear-time algorithm for gaussian and non-gaussian trait evolution models. *Syst. Biol.* 63, 397-408. 10.1093/sysbio/syu005 24500037

[JEB250347C28] Janoo, A. and Gasc, J.-P. (1992). High speed motion analysis of the predatory strike and fluorographic study of oesophageal deglutition in *Vipera ammodytes*: more than meets the eye. *Amphib-Reptilia* 13, 315-325. 10.1163/156853892X00021

[JEB250347C29] Jestrzemski, D. and Kuzyakova, I. (2019). Morphometric characteristics and seasonal proximity to water of the Cypriot bluntnosed viper *Macrovipera lebetina lebetina* (Linnaeus, 1758). *J. Venom. Anim. Toxins Incl. Trop. Dis.* 24, 42. 10.1186/s40409-018-0175-6 PMC6307313 30607143

[JEB250347C30] Kardong, K. V. (1986). The predatory strike of the rattlesnake: when things go amiss. *Copeia* 1986, 816-820. 10.2307/1444969

[JEB250347C31] Kardong, K. V. and Bels, V. L. (1998). Rattlesnake strike behavior: kinematics. *J. Exp. Biol.* 201, 837-850. 10.1242/jeb.201.6.837 9464964

[JEB250347C32] Konow, N. and Sanford, C. P. (2008). Biomechanics of a convergently derived prey-processing mechanism in fishes: evidence from comparative tongue bite apparatus morphology and raking kinematics. *J. Exp. Biol.* 211, 3378-3391. 10.1242/jeb.023564 18931311

[JEB250347C33] LaDuc, T. J. (2002). Does a quick offense equal a quick defense? Kinematic comparisons of predatory and defensive strikes in the western diamond-backed rattlesnake (*Crotalus atrox*). In *Biology of the Vipers* (ed. G. W. Schuett, M. Höggren, M. E. Douglas and H. W. Greene), pp. 267-278. Eagle Mountain Publishers.

[JEB250347C34] Lee, T. H. (2005). Ecological patterns of distribution on gradients of elevation and species diversity of snakes in southern Taiwan. *Amphib-Reptilia* 26, 325-332. 10.1163/156853805774408522

[JEB250347C35] Leenders, T. (2019). *Reptiles of Costa Rica: a Field Guide*. Cornell University Press.

[JEB250347C36] Lester, H. M. (1955). How we photographed a rattler's strike. *Anim. Kingdom* 58, 116-123.

[JEB250347C37] Lomonte, B., Escolano, J., Fernández, J., Sanz, L., Angulo, Y., Gutiérrez, J. M. and Calvete, J. J. (2008). Snake venomics and antivenomics of the arboreal neotropical pitvipers *Bothriechis lateralis* and *Bothriechis schlegelii*. *J. Proteome Res.* 7, 2445-2457. 10.1021/pr8000139 18444672

[JEB250347C38] Luiselli, L. (2006). Site occupancy and density of sympatric Gaboon viper (*Bitis gabonica*) and nose-horned viper (*Bitis nasicornis*). *J. Trop. Ecol.* 22, 555-564. 10.1017/S0266467406003397

[JEB250347C39] Luiselli, L. and Angelici, F. M. (2000). Ecological relationships in two Afrotropical cobra species (*Naja melanoleuca* and *Naja nigricollis*). *Can. J. Zool.* 78, 191-198. 10.1139/z99-200

[JEB250347C40] Luu, V. Q., Nguyen, T. Q., Lehmann, T., Bonkowski, M. and Ziegler, T. (2015). New records of the horned pitviper, *Protobothrops cornutus* (Smith, 1930) (Sepentes: Viperidae), from Vietnam with comments on morphological variation. *Herpetol. Notes* 8, 149-152.

[JEB250347C41] Martínez-Freiría, F., Crochet, P.-A., Fahd, S., Geniez, P., Brito, J. C. and Velo-Antón, G. (2017). Integrative phylogeographical and ecological analysis reveals multiple Pleistocene refugia for Mediterranean Daboia vipers in north-west Africa. *Biol. J. Linn. Soc.* 122, 366-384. 10.1093/biolinnean/blx038

[JEB250347C42] Martins, M., Araujo, M. S., Sawaya, R. J. and Nunes, R. (2001). Diversity and evolution of macrohabitat use, body size and morphology in a monophyletic group of Neotropical pitvipers (*Bothrops*). *J. Zool.* 254, 529-538. 10.1017/S0952836901001030

[JEB250347C43] Mehta, R. S. and Wainwright, P. C. (2007). Biting releases constraints on moray eel feeding kinematics. *J. Exp. Biol.* 210, 495-504. 10.1242/jeb.02663 17234619

[JEB250347C44] Moon, B. R., Penning, D. A., Segall, M. and Herrel, A. (2019). Feeding in Snakes: Form, Function, and Evolution of the Feeding System. In *Feeding in Vertebrates: Evolution, Morphology, Behavior, Biomechanics* (ed. V. Bels and I. Q. Whishaw), pp. 527-574. Springer.

[JEB250347C45] Muntean, M. E., Ghira, I. V. and Marosi, B. A. (2009). Comparative study on the chemical perception and prey odor preference in newborns of *Coluber* and *Viper* snake species. *Herpetol. Romanica* 3, 41-45.

[JEB250347C46] Nasoori, A., Shahbazzadeh, D., Tsubota, T. and Young, B. A. (2016). The defensive behaviour of *Naja oxiana*, with comments on the visual displays of cobras. *Herpetol. Bull.* 138, 13-17.

[JEB250347C47] Naulleau, G. and Bonnet, X. (1995). Reproductive ecology, body fat reserves and foraging mode in females of two contrasted snake species: *Vipera aspis* (terrestrial, viviparous) and *Elaphe longissima* (semi-arboreal, oviparous). *Amphib-Reptilia* 16, 37-46. 10.1163/156853895X00172

[JEB250347C48] Oliveira, M. E. and Martins, M. (2001). When and where to find a pitviper: activity patterns and habitat use of the lancehead, *Bothrops atrox*, in central Amazonia, Brazil. *Herpetol. Nat. History* 8, 101-110.

[JEB250347C49] Penning, D. A., Sawvel, B. and Moon, B. R. (2016). Debunking the viper's strike: harmless snakes kill a common assumption. *Biol. Lett.* 12, 20160011. 10.1098/rsbl.2016.0011 26979562 PMC4843225

[JEB250347C50] Penning, D. A., Sawvel, B. and Moon, B. R. (2020). The scaling of terrestrial striking performance in western ratsnakes (*Pantherophis obsoletus*). *J. Exp. Zool. A Ecol. Integr. Physiol.* 333, 96-103. 10.1002/jez.2328 31625282

[JEB250347C51] Porras, L. W., Wilson, L. D., Schuett, G. W. and Reiserer, R. S. (2013). A taxonomic reevaluation and conservation assessment of the common cantil, *Agkistrodon bilineatus* (Squamata: Viperidae): a race against time. *Amphibian & Reptile Conservation* 7, 48-73.

[JEB250347C52] Putman, B. J. and Clark, R. W. (2017). Behavioral thermal tolerances of free-ranging rattlesnakes (*Crotalus oreganus*) during the summer foraging season. *J. Therm. Biol.* 65, 8-15. 10.1016/j.jtherbio.2017.01.012 28343580

[JEB250347C53] Richards, D. P., Barlow, A. and Wüster, W. (2012). Venom lethality and diet: differential responses of natural prey and model organisms to the venom of the saw-scaled vipers (*Echis*). *Toxicon* 59, 110-116. 10.1016/j.toxicon.2011.10.015 22079297

[JEB250347C54] Ryerson, W. G. (2020). Ontogeny of strike performance in ball pythons (*Python regius*): a three-year longitudinal study. *Zoology* 140, 125780. 10.1016/j.zool.2020.125780 32289748

[JEB250347C55] Ryerson, W. G. and Tan, W. (2017). Strike kinematics and performance in juvenile ball pythons (*Python regius*). *J. Exp. Zool. A Ecol. Integr. Physiol.* 327, 453-457. 10.1002/jez.2131 29356394

[JEB250347C56] Santos, X., Llorente, G., Pleguezuelos, J., Brito, J., Fahd, S. and Parellada, X. (2007). Variation in the diet of the Lataste's viper *Vipera latastei* in the Iberian Peninsula: seasonal, sexual and size-related effects. *Anim. Biol.* 57, 49-61. 10.1163/157075607780001998

[JEB250347C57] Sawant, N. S., Jadhav, T. D. and Shyama, S. (2010). Habitat suitability, threats and conservation strategies of hump-nosed pit viper *Hypnale hymnal* Merrem (Reptilia: Viperidae) found in Western Ghats, Goa, India. *J. Threatened Taxa* 2, 1261-1267. 10.11609/JoTT.o2490.1261-7

[JEB250347C58] Shine, R. (1991). Strangers in a strange land: ecology of the Australian colubrid snakes. *Copeia* 1991, 120-131. 10.2307/1446254

[JEB250347C59] Shine, R., Spencer, C. L. and Keogh, J. S. (2014). Morphology, reproduction and diet in Australian and Papuan death adders (*Acanthophis*, Elapidae). *PLoS ONE* 9, e94216. 10.1371/journal.pone.0094216 24718608 PMC3981772

[JEB250347C60] So, K. K., Wainwright, P. and Bennett, A. (1992). Kinematics of prey processing in *Chamaeleo jacksonii*: conservation of function with morphological specialization. *J. Zool.* 226, 47-64. 10.1111/j.1469-7998.1992.tb06126.x

[JEB250347C61] Sutton, W. B., Wang, Y., Schweitzer, C. J. and Mcclure, C. J. (2017). Spatial ecology and multi-scale habitat selection of the copperhead (*Agkistrodon contortrix*) in a managed forest landscape. *For. Ecol. Manag.* 391, 469-481. 10.1016/j.foreco.2017.02.041

[JEB250347C62] Title, P. O., Singhal, S., Grundler, M. C., Costa, G. C., Pyron, R. A., Colston, T. J., Grundler, M. R., Prates, I., Stepanova, N. and Jones, M. E. (2024). The macroevolutionary singularity of snakes. *Science* 383, 918-923. 10.1126/science.adh2449 38386744

[JEB250347C63] Van Riper, W. (1953). How a rattlesnake strikes. *Sci. Am.* 189, 100-103. 10.1038/scientificamerican1053-100

[JEB250347C64] Venesky, M. D., Wassersug, R. J., Jorgensen, M. E., Riddle, M. and Parris, M. J. (2011). Comparative feeding kinematics of temperate pond-dwelling tadpoles (Anura, Amphibia). *Zoomorphology* 130, 31-38. 10.1007/s00435-011-0119-y

[JEB250347C65] Vincent, S. E., Herrel, A. and Irschick, D. J. (2005). Comparisons of aquatic versus terrestrial predatory strikes in the pitviper, *Agkistrodon piscivorus*. *J. Exp. Zool. A Comp. Exp. Biology* 303, 476-488. 10.1002/jez.a.179 15880763

[JEB250347C66] Wainwright, P. C., Kraklau, D. M. and Bennett, A. F. (1991). Kinematics of tongue projection in *Chamaeleo oustaleti*. *J. Exp. Biol.* 159, 109-133. 10.1242/jeb.159.1.109

[JEB250347C67] Wasko, D. K. and Sasa, M. (2009). Activity patterns of a neotropical ambush predator: spatial ecology of the Fer–de–lance (*Bothrops asper*, Serpentes: Viperidae) in Costa Rica. *Biotropica* 41, 241-249. 10.1111/j.1744-7429.2008.00464.x

[JEB250347C68] Webb, J. K. and Shine, R. (1998). Thermoregulation by a nocturnal elapid snake (*Hoplocephalus bungaroides*) in southeastern Australia. *Physiol. Zool.* 71, 680-692. 10.1086/515979 9798255

[JEB250347C69] Whitford, M. D., Freymiller, G. A., Higham, T. E. and Clark, R. W. (2020). The effects of temperature on the defensive strikes of rattlesnakes. *J. Exp. Biol.* 223, jeb223859. 10.1242/jeb.223859 32561628

[JEB250347C70] Wickham, H. (2016). *ggplot2: Elegant Graphics for Data Analysis*. Springer.

[JEB250347C71] Wickum, B., Thuen, B. and Deufel, A. (2007). Burrowing in the coral cobra (*Aspidelaps lubricus*). *Proc. N. D. Acad. Sci.* 61, 22-23.

[JEB250347C72] Yilmaz, M. and Meister, M. (2013). Rapid innate defensive responses of mice to looming visual stimuli. *Curr. Biol.* 23, 2011-2015. 10.1016/j.cub.2013.08.015 24120636 PMC3809337

[JEB250347C73] Young, B. A. (2010). How a heavy–bodied snake strikes quickly: high–power axial musculature in the puff adder (*Bitis arietans*). *J. Exp. Zool. A Ecol. Genet. Physiol.* 313, 114-121. 10.1002/jez.579 20039332

[JEB250347C74] Young, B. A., Blair, M., Zahn, K. and Marvin, J. (2001). Mechanics of venom expulsion in *Crotalus*, with special reference to the role of the fang sheath. *Anat. Rec.* 264, 415-426. 10.1002/ar.10015 11745096

[JEB250347C75] Youngman, N. J., Chowdhury, A., Zdenek, C. N., Coster, K., Sundman, E., Braun, R. and Fry, B. G. (2021). Utilising venom activity to infer dietary composition of the Kenyan horned viper (*Bitis worthingtoni*). *Comp. Biochem. Physiol. C Toxicol. Pharmacol.* 240, 108921. 10.1016/j.cbpc.2020.108921 33122136

