# Supplementary material for: Kinematics of strikes in venomous snakes
Source: J Exp Biol. 2025 Oct 23;228(20):jeb250347. doi: 10.1242/jeb.250347 (PMC12582407; doi:10.1242/jeb.250347)
Supplement: Supplementary information [file jexbio-228-250347-s1.pdf]

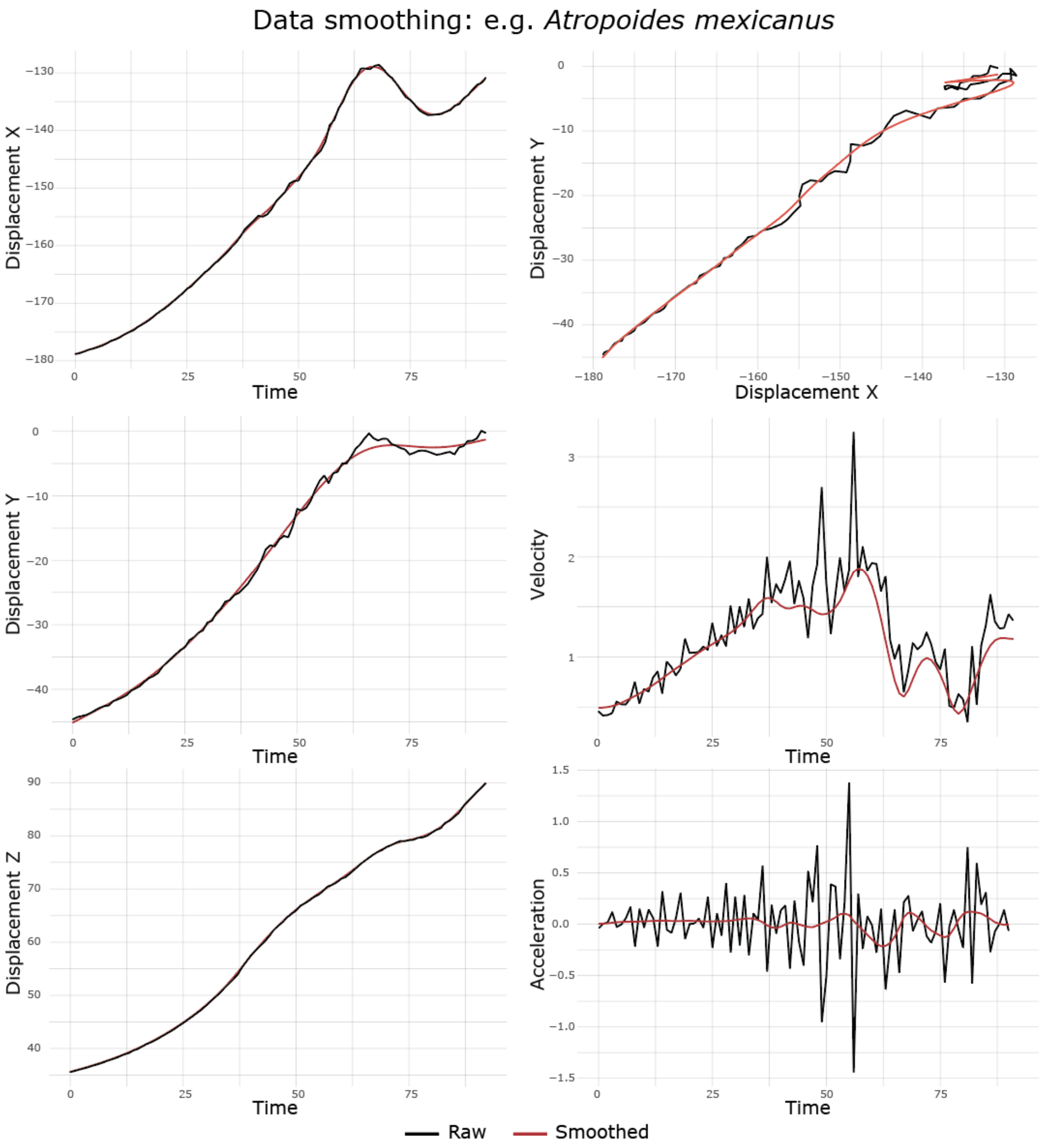

Fig. S1. Time series (in frames at 1000 frames per second) for X, Y, and Z coordinates, and X vs Y, for eye position of *Atropoides mexicanus* during prey strike. Raw data collected from video (black) and data smoothed using cubic spline (red). Velocity (m s^{-1}) and acceleration (m s^{-2}) calculated from raw data (black) and smoothed data (red).

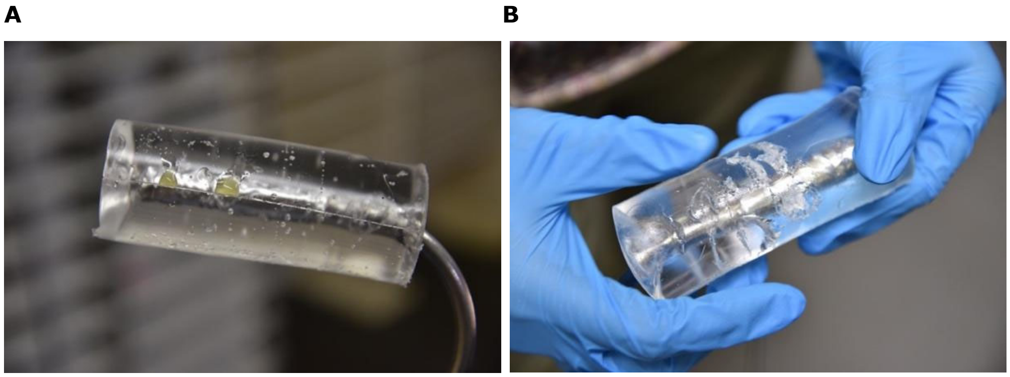

Fig. S2. Gelatine prey after snake bite. Droplets of venom were often left behind at the injection sites (A). The colubrid snake *Toxicodryas pulverulenta* uses its rear fangs to slice, creating large crescent-shaped ‘wounds’ in the gel (B).

Journal of Experimental Biology • Supplementary information

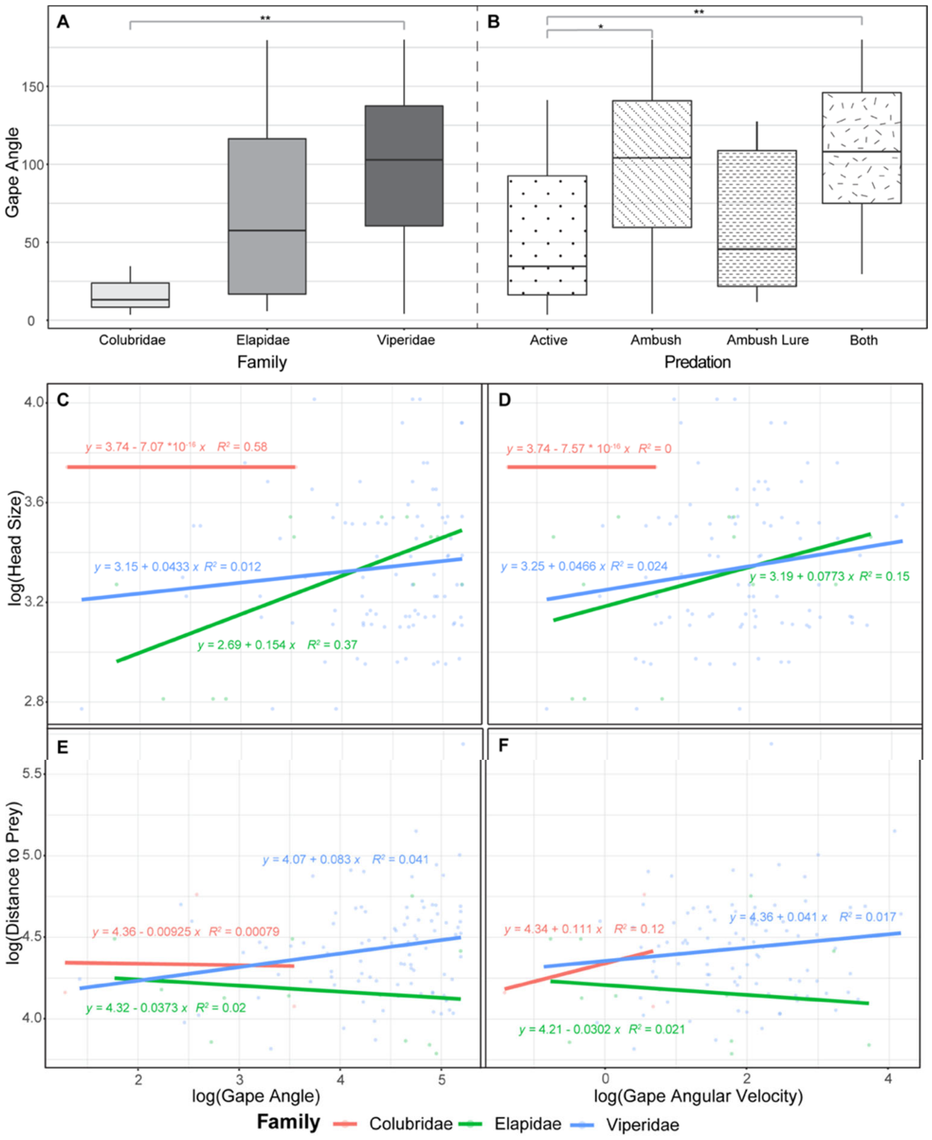

Fig. S3. Interpretation results gape angle and angular velocity of gape opening. (A) Boxplot showing difference in gape angle across family. (B) Boxplot showing difference in gape angle across predation style. Regression of head size and gape angle (C) and angular velocity (D). Regression of Distance to Prey and gape angle (E) and angular velocity (F).

Journal of Experimental Biology • Supplementary information

Table S1. List of recorded species and their preferences for diet, habitat, predation style and biorhythm.

Genus	Species	Diet	Habitat	Predation	Biorhythm
			**Colubridae**		
Boiga	*dendrophila*	Generalist	Arboreal	Active	Nocturnal
			**Elapidae**		
Acanthophis	*rugosus*	Ontogenetic	Terrestrial	Ambush	Diurnal
Aspidelaps	*lubricus*	Scaly	Sanddwelling	Active	Nocturnal
Naja	*melanoleuca*	Generalist	Semi-aquatic	Active	Diurnal
Walterinnesia	*aegyptia*	Scaly	Terrestrial	Active	Nocturnal
			**Viperidae**		
Agkistrodon	*contortrix*	Ontogenetic	Terrestrial	Ambush	Seasonal
	taylori	Soft	Terrestrial	Ambush	Seasonal
Atropoides	*mexicanus*	Ontogenetic	Terrestrial	Ambush	Nocturnal
Bitis	*nasicornis*	Ontogenetic	Terrestrial	Ambush	Nocturnal
Bothriechis	*schlegelii*	Generalist	Arboreal	Ambush	Nocturnal
Bothrops	*asper*	Ontogenetic	Terrestrial	Ambush	Nocturnal
	atrox	Ontogenetic	Terrestrial	Ambush	Nocturnal
	taeniatus	Generalist	Arboreal	Lure	Nocturnal
Cerastes	*cerastes*	Generalist	Sanddwelling	Both	Nocturnal
Crotalus	*atrox*	Soft	Terrestrial	Both	Nocturnal
	lepidus	Scaly	Terrestrial	Ambush	Nocturnal
	oreganus	Generalist	Terrestrial	Both	Seasonal
	scutulatus	Soft	Terrestrial	Both	Seasonal
Daboia	*palaestina*	Ontogenetic	Terrestrial	Ambush	Nocturnal
Deinagkistrodon	*acutus*	Soft	Terrestrial	Ambush	Nocturnal
Echis	*carinatus*	Arthropod	Terrestrial	Ambush	Nocturnal
	leucogaster	Generalist	Terrestrial	Ambush	Nocturnal
	ocellatus	Generalist	Terrestrial	Ambush	Seasonal
Eristicophis	*macmahoni*	Generalist	Sanddwelling	Both	Nocturnal
Hypnale	*hypnale*	Scaly	Terrestrial	Lure	Nocturnal
Macrovipera	*lebetina*	Ontogenetic	Terrestrial	Ambush	Seasonal
Porthidium	*ophryomegas*	Ontogenetic	Terrestrial	Ambush	Seasonal
Proatheris	*superciliaris*	Generalist	Terrestrial	Ambush	Seasonal
Protobothrops	*cornutus*	Ontogenetic	Semi-arboreal	Ambush	Nocturnal
	jerdonii	Soft	Semi-arboreal	Ambush	Seasonal
	mucrosquamatus	Generalist	Terrestrial	Ambush	Nocturnal
Trimeresurus	*albolabris*	Soft	Semi-arboreal	Active	Nocturnal
	trigonocephalus	Generalist	Arboreal	Ambush	Nocturnal
Vipera	*ammodytes*	Ontogenetic	Terrestrial	Ambush	Seasonal
	aspis	Ontogenetic	Terrestrial	Ambush	Seasonal
	latastei	Ontogenetic	Terrestrial	Ambush	Seasonal

Journal of Experimental Biology • Supplementary information

Table S2. List of individuals included in this study with the number of strikes filmed and the three videos that were included in the kinematical analysis. NA = not applicable.

Genus	Species	ID	Videos for kinematics	Total # videos
Boiga	*dendrophila*	STV1510-0001	2, 3, 4	5
Acanthophis	*rugosus*	STV1806-0022	1, 2, 3	3
Aspidelaps	*lubricus*	STV1808-0007	1, 2, 3	3
Naja	*melanoleuca*	STV1438-0004	3, 4, 5	6
Walterinnesia	*aegyptia*	STV1836-0022	1, 2, 3	3
Agkistrodon	*contortrix*	STV1481-0025	6, 7, 8	8
	taylori	STV1655-0013	1, 3, 5	6
Atropoides	*mexicanus*	STV1710-0002	1, 2, 4	4
Bitis	*nasicornis*	STV1757-0027	2, 3, 4	6
Bothriechis	*schlegelii*	STV1520-0006	2, 3, 4	5
Bothrops	*asper*	STV1633-0011	1, 2, 3	4
	atrox	STV1476-0128	1, 2, 4	4
	taeniatus	STV1612-0004	3, 4, 5	5
Cerastes	*cerastes*	STV1823-0023	1, 2, 3	3
Crotalus	*atrox*	STV1637-0021	1, 2, 5	6
	lepidus	STV1513-0001	1, 2, 5	5
	oreganus	STV1487-0004	1, 2, 3	3
	scutulatus	STV1638-0012	1, 2, 3	4
Daboia	*palaestinae*	STV1560-0048	2, 3, 4	4
Deinagkistrodon	*acutus*	STV1747-0001	2, 3, 4	5
Echis	*carinatus*	STV1717-0031	1, 2, 3	3
	leucogaster	STV1627-0073	1, 2, 3	3
	ocellatus	STV1545-0028	1, 2, 3	3
Eristicophis	*macmahoni*	STV1634-0007	1, 2, 4	4
Hypnale	*hypnale*	STV1431-0002	1, 3, 4	4
Macrovipera	*lebetina*	STV1608-0010	6, 7, 8	8
Porthidium	*ophryomegas*	STV1731-0001	1, 2, 3	3
Proatheris	*superciliaris*	STV1665-0020	1, 7, 8	8
Protobothrops	*cornutus*	STV1924-0014	1, 4, 5	7
	jerdonii	STV1925-0006	1, 2, 4	8
	mucrosquamatus	STV1926-0006	3, 4, 5	5
Trimeresurus	*albolabris*	STV1607-0003	2, 4, 5	5
	trigonocephalus	STV1518-0020	1, 2, 3	3
Toxicodryas	*pulverulenta*	STV1810-0001	NA	3
Vipera	*ammodytes*	STV1447-0133	2, 3, 6	6
	aspis	STV1622-0015	3, 4, 6	7
	latastei	STV1471-0001	1, 2, 4	4

Table S3. Results from phylolm analysis (Phylogenetic generalised least squares regression and phylogenetic signal) of kinematic variables against predictor variables for Viperidae. Significance indicated in bold.

Available for download at
https://journals.biologists.com/jeb/article-lookup/doi/10.1242/jeb.250347#supplementary-data

Table S4. Results from phylolm analysis (Phylogenetic generalised least squares regression and phylogenetic signal) of head size against predictor variables for Viperidae. Significance indicated in bold.

Available for download at https://journals.biologists.com/jeb/article-lookup/doi/10.1242/jeb.250347#supplementary-data

Table S5. Results from phylolm analysis (Phylogenetic generalised least squares regression and phylogenetic signal) of Start Distance against kinematic variables for Viperidae. Significance indicated in bold.

Available for download at https://journals.biologists.com/jeb/article-lookup/doi/10.1242/jeb.250347#supplementary-data

Table S6. Results from phylolm analysis (Phylogenetic generalised least squares regression and phylogenetic signal) of fang kinematic variables against predictor variables for Viperidae. Significance indicated in bold.

Available for download at https://journals.biologists.com/jeb/article-lookup/doi/10.1242/jeb.250347#supplementary-data

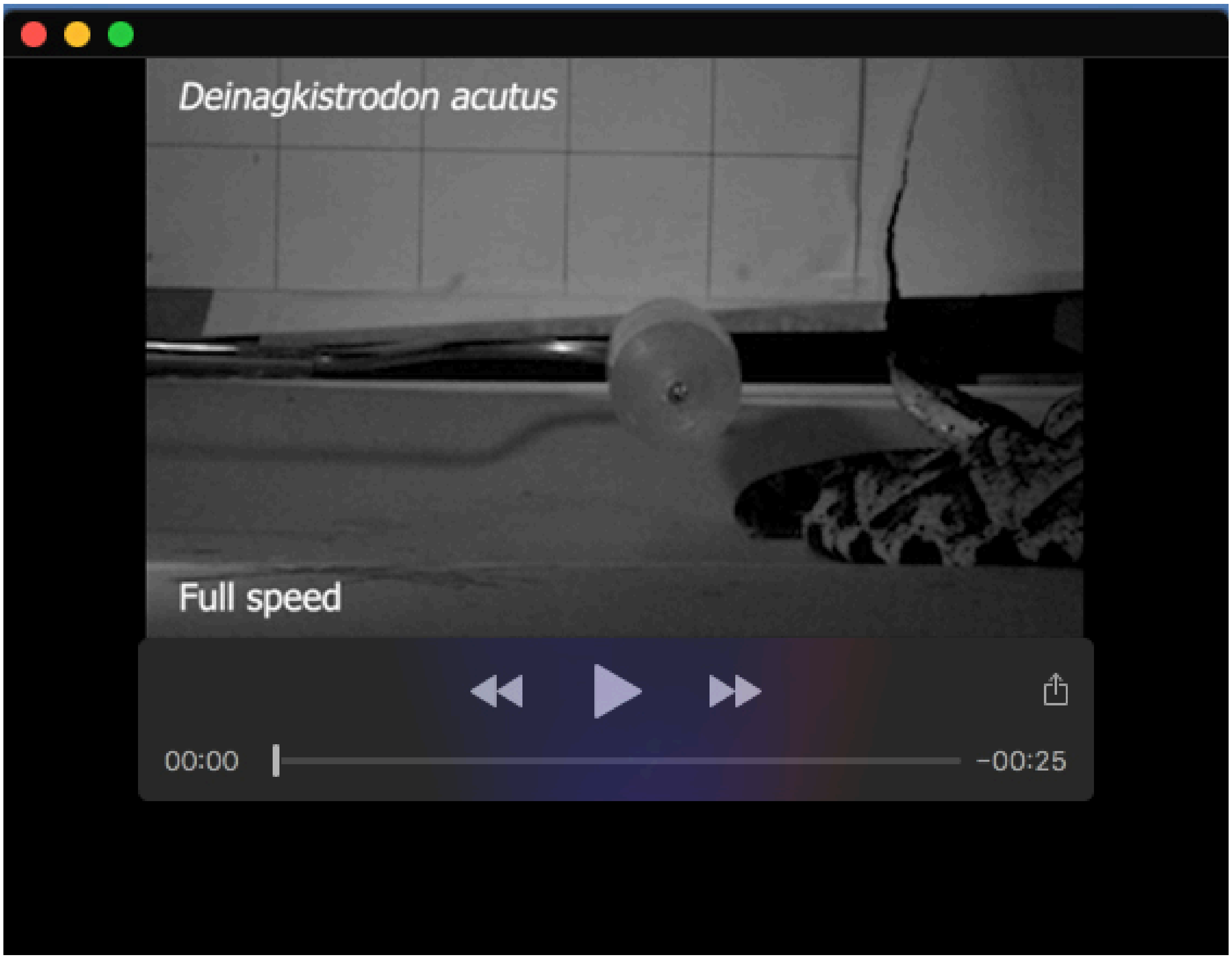

Movie 1. Reconstruction of 3D kinematics of the feeding strike of viper from two views. Sequence 1: full speed strike by *Deinagkistrodon acutus* (ID STV1747-0001) (1000 frames per second), completed in 0.11 seconds. Sequence 2: snake strike at 3% speed (30 frames per second). Sequence 3: two simultaneous views of snake strike from 90° (top, video 2A) and 60° (bottom, video 2B) with landmarks on snake and gel prey placed on both views. Sequence 4: landmark movement in 3D space reconstructed from two views. Line colours match landmark colours in Sequence 3, and jaw landmarks connected with pink lines to show jaw opening and closing.

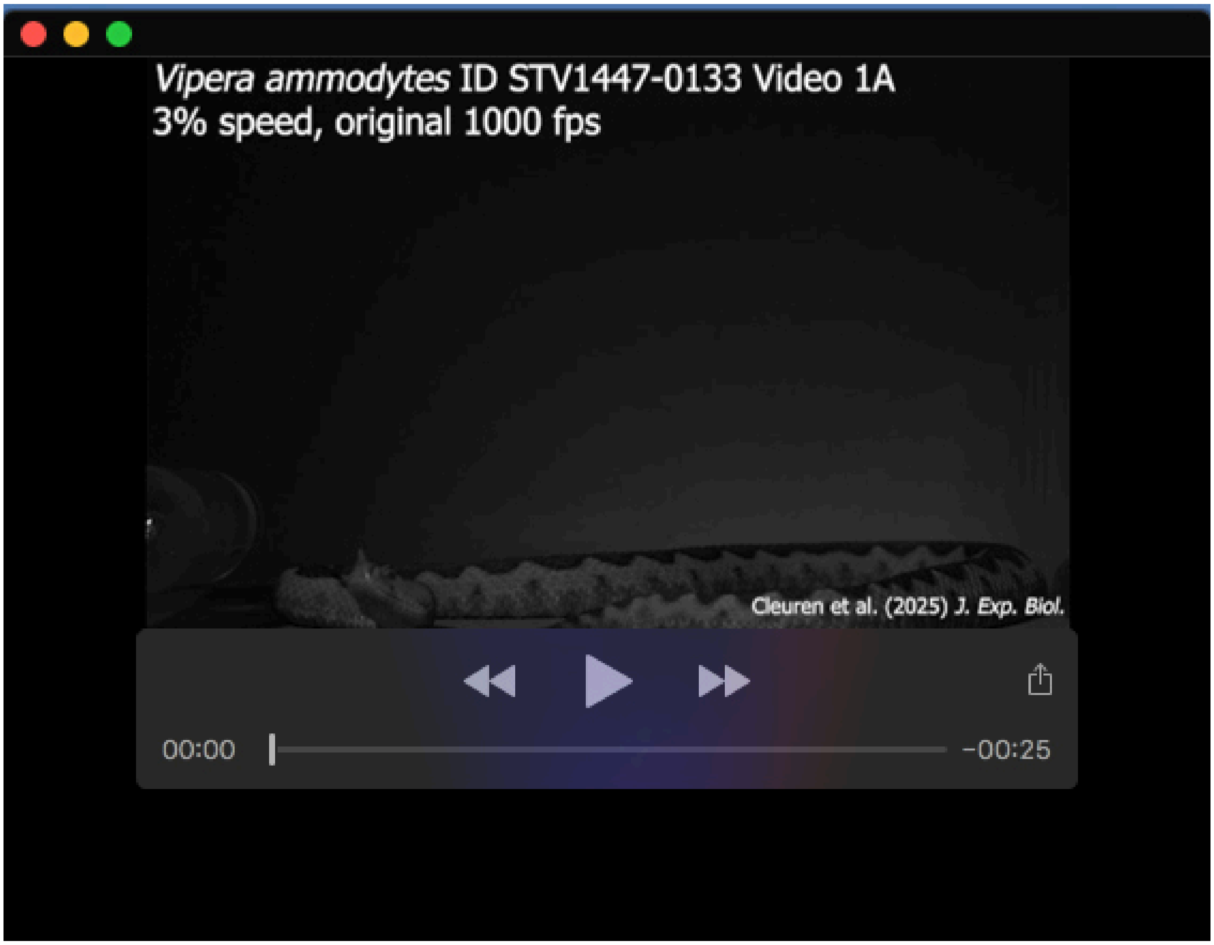

Movie 2. Example of a feeding strike by a viperid snake. After initial bite, fangs are 'walked' further onto the prey, and injected venom squirts out of the gel before releasing gel. Feeding strike frames shown in Fig. 5. *Vipera ammodytes* (ID STV1447-0133) video 1A (90º camera). Recorded at 1000 frames per second and played back at 30 frames per second (3% speed).

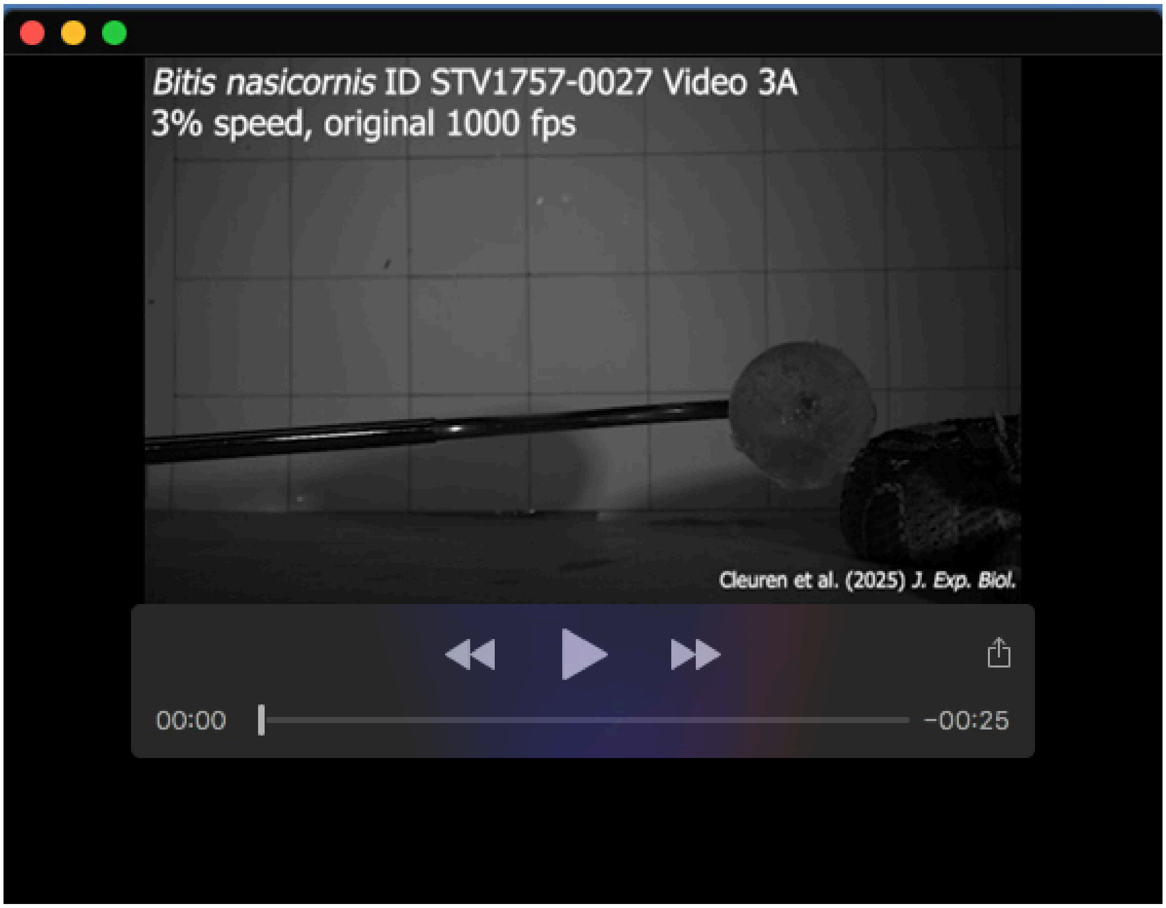

Movie 3. Example of a feeding strike by a viperid snake, where venom drips out of fang before fang penetrates gel. *Bitis nasicornis* (ID STV1757- 0027) video 3A (90º camera). Recorded at 1000 frames per second and played back at 30 frames per second (3% speed).

Journal of Experimental Biology • Supplementary information

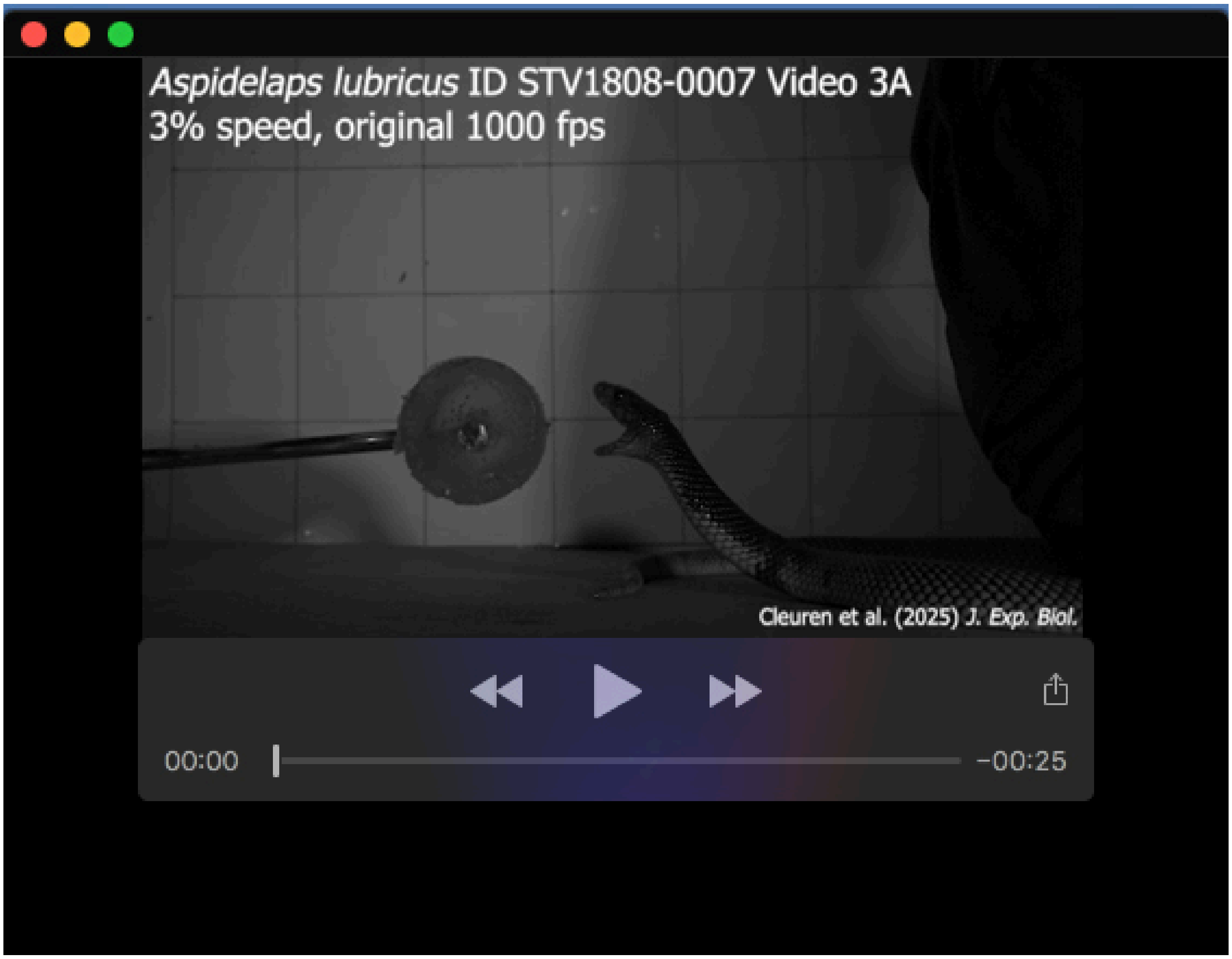

Movie 4. Example of a feeding strike by an elapid snake, showing repeated biting action. *Aspidelaps lubricus* (ID STV1808-0007) video 3A (90º camera). Recorded at 1000 frames per second and played back at 30 frames per second (3% speed).

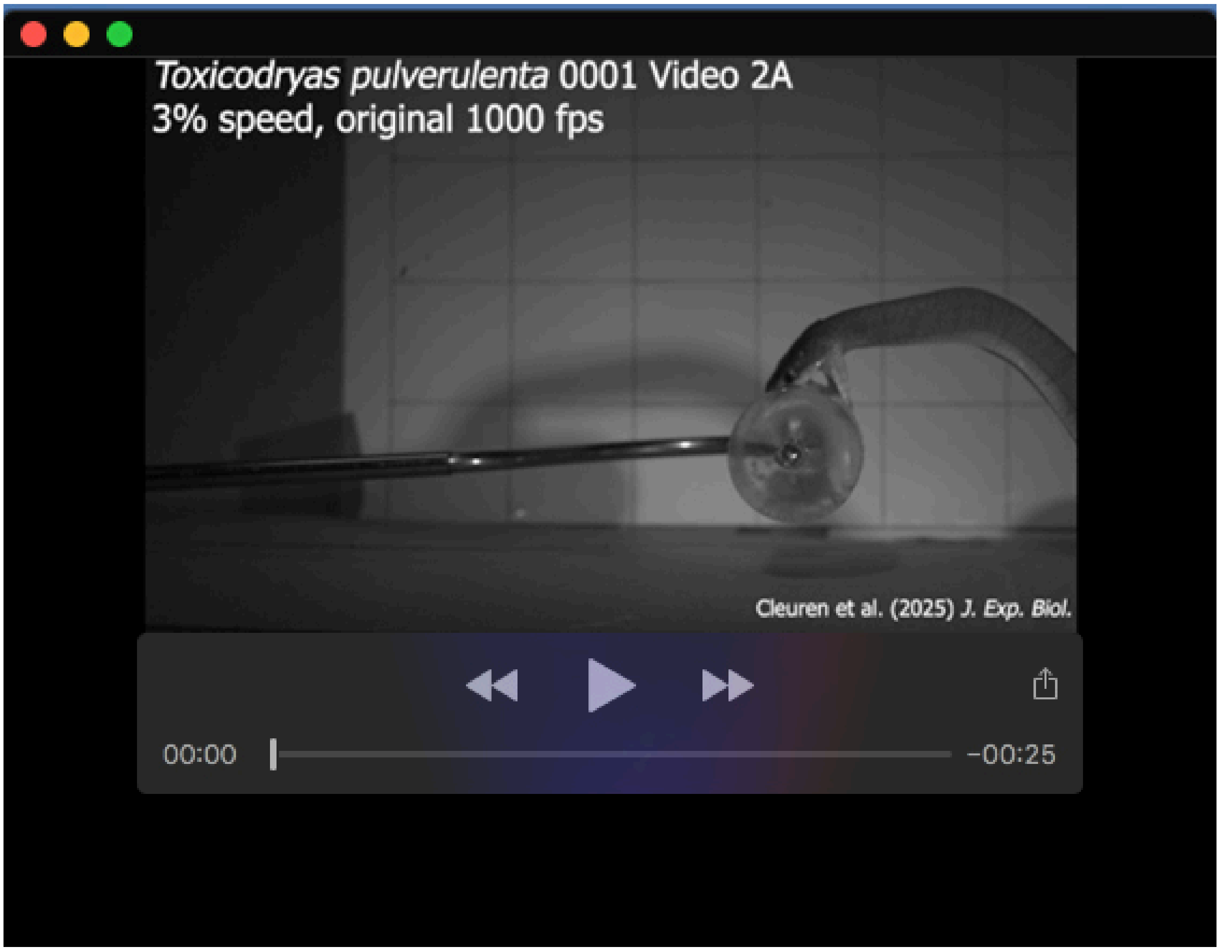

Movie 5. Example of a feeding strike by a colubrid snake, showing dragging of the maxilla across the prey surface. *Toxicodryas pulverulenta* (ID STV1810-0001) video 2A (90º camera). Recorded at 1000 frames per second and played back at 30 frames per second (3% speed).

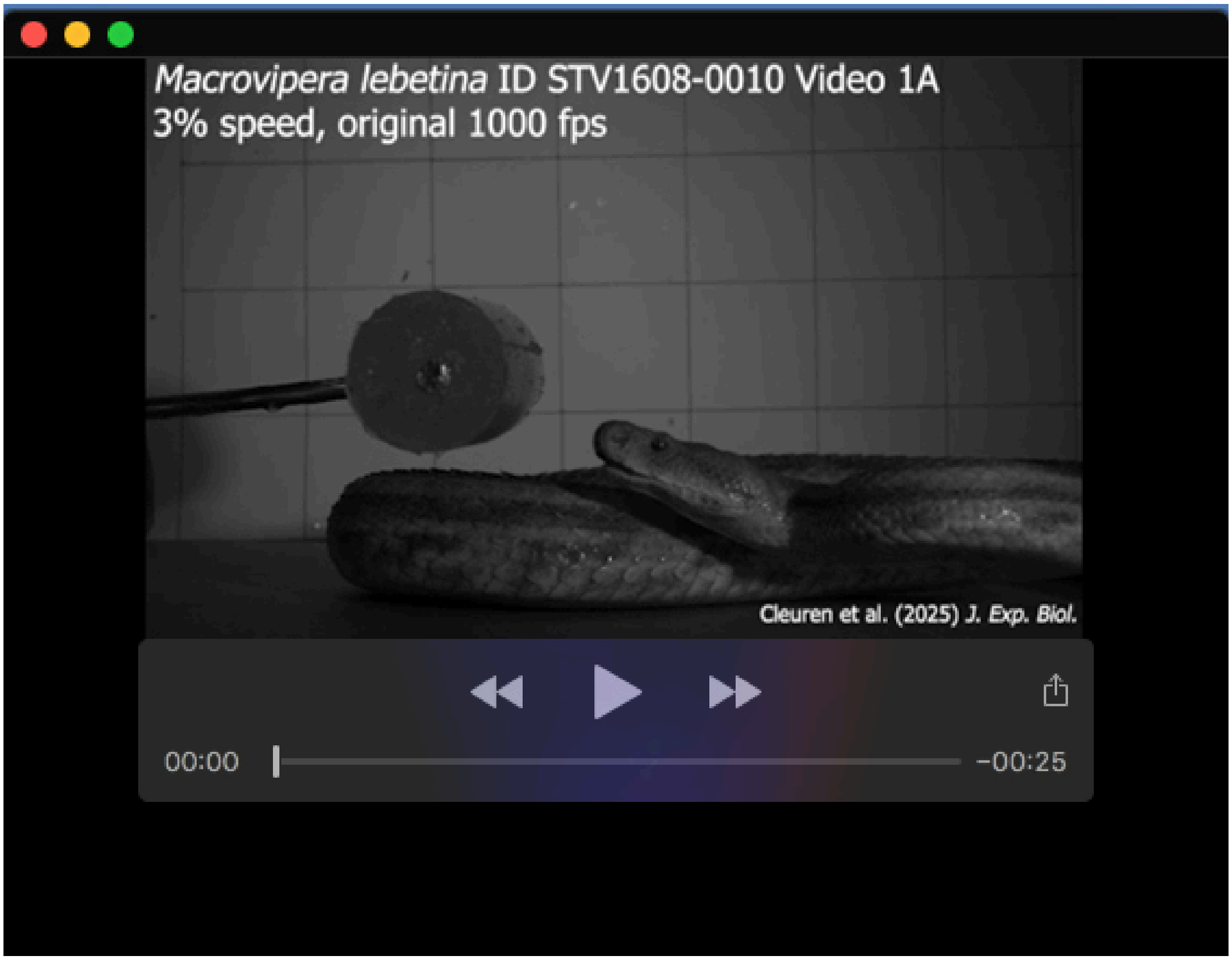

Movie 6. A feeding strike where the snake hit its right fang on the gel and the fang broke off. *Macrovipera lebetina* (ID STV1608-0010) video 1A (90º camera). Recorded at 1000 frames per second and played back at 30 frames per second (3% speed).

Journal of Experimental Biology • Supplementary information
